# Spatial autocorrelation with environmental factors related to tuberculosis prevalence in Nepal, 2020–2023

**DOI:** 10.1186/s40249-025-01283-y

**Published:** 2025-03-03

**Authors:** Roshan Kumar Mahato, Kyaw Min Htike, Alex Bagas Koro, Rajesh Kumar Yadav, Vijay Sharma, Alok Kafle, Suvash Chandra Ojha

**Affiliations:** 1 Faculty of Public Health, Khon Kaen University, Khon Kaen, Thailand; 2 Department of Public Health, LA GRANDEE International College, Pokhara University, Pokhara, Nepal; 3 Kathmandu University School of Medical Sciences, Dhulikhel, Nepal; 4 Department of Tropical Medicine, Faculty of Medicine, Khon Kaen University, Khon Kaen, Thailand; 5 Department of Infectious Diseases, The Affiliated Hospital of Southwest Medical University, Luzhou, 646000 China

**Keywords:** Tuberculosis, Environmental factors, Local indicators of spatial association, Regression analysis, Nepal

## Abstract

**Background:**

Despite global efforts to reduce tuberculosis (TB) incidence, Nepal remains burdened by approximately 70,000 new cases annually, with an incidence rate of 229 per 100,000 people in 2022. This study investigated the geographic patterns of TB notifications in Nepal from fiscal year 2020 to 2023, focusing on environmental determinants such as land surface temperature (LST), urbanization, precipitation and cropland coverage.

**Methods:**

This study examined the spatial association between environmental factors and TB prevalence in Nepal at the district level, utilizing Geographic Information System (GIS) techniques, bivariate Local Indicators of Spatial Association (LISA) and spatial regression analyses. The tuberculosis prevalence data were obtained from the National Tuberculosis Control Center (NTCC) Nepal for the fiscal years (FY) 2020–2023.

**Results:**

Over the three fiscal years, high TB prevalence consistently clustered in districts such as Banke, Parsa, and Rautahat, while low prevalence areas included Mustang and Kaski. Significant positive spatial autocorrelation was found between environmental factors and TB prevalence. Moran’s *I* values were as follows: for LST (day), 0.379, 0.424, and 0.423; for LST (night), 0.383, 0.420, and 0.425; for cropland coverage, 0.325, 0.339, and 0.373; for urbanization, 0.197, 0.245, and 0.246; and for precipitation, 0.222, 0.349, and 0.104 across FY 2020–2021, FY 2021–2022 and FY 2022–2023, respectively. Regression analyses, including Ordinary Least Squares (OLS), Spatial Lag Model (SLM), and Spatial Error Model (SEM), demonstrated that Land Surface Temperature Night (LSTN), urbanization, and precipitation significantly influenced TB prevalence, explaining up to 72.1% of the variance in FY 2021–2022 (R^2^: 0.721).

**Conclusions:**

Environmental factors significantly influence the spatial distribution of TB in Nepal. This underscores the importance of integrating disease management strategies with environmental health policies in effectively addressing TB prevalence.

**Graphical Abstract:**

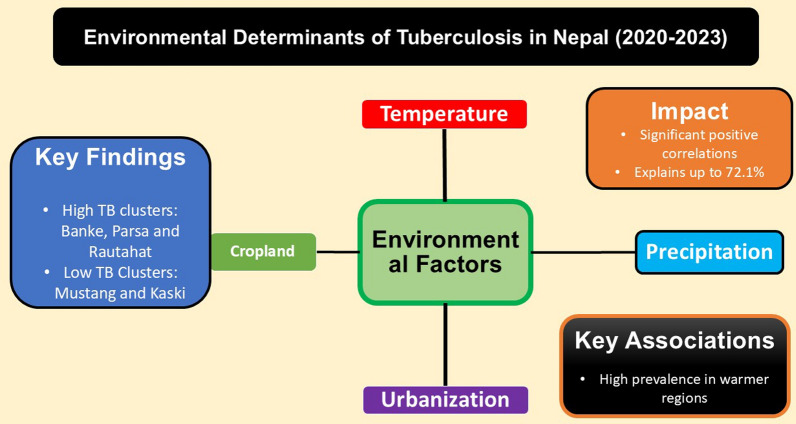

## Background

Tuberculosis (TB), historically the leading cause of death from a single infectious agent, continues to face challenges in access to essential services due to the significant health, social and economic disruptions [1]. The Global Tuberculosis Report 2024 highlighted an increase in newly diagnosed TB cases, from 7.5 million in 2022 to 8.2 million in 2023, surpassing pre-pandemic levels [2]. Despite reductions in prevalence over the past decade, TB remains a critical threat, particularly in low- and middle-income (LMIC) countries [3]. In 2022, an estimated 410,000 people globally developed multidrug-resistant or rifampicin-resistant TB (MDR/RR-TB), complicating treatment and management efforts [4]. HIV coinfection in high-burden settings significantly heightens the risk of active TB, accelerating the progression to AIDS. This necessitates integrated management strategies that combine anti-TB treatment with antiretroviral therapy (ART) while addressing comorbidities [5].

At the regional level, South Asia’s TB Control Program prioritizes cross-border cooperation to tackle shared challenges, including population migration, drug resistance, and limited healthcare access [6]. In 2022, the region accounted for approximately 38% of global MDR/RR-TB cases, with an estimated 170,000 cases, though only 74,300 received treatment. Despite these challenges, the overall incidence of TB in South Asia decreased from 269 to 234 cases per 100,000 people [7].

Nepal grapples with a significant burden of tuberculosis. In 2022, the country reported approximately 70,000 new cases and an incidence rate of 229 per 100,000 people [8]. Uneven case distribution, diagnostic delays, social stigma, and limited healthcare access impede effective TB control. Diverse environmental and socio-economic factors influence TB prevalence across Nepal, underscoring its public health significance [9]. Globally, efforts to reduce TB incidence have successfully decreased new cases from 163 to 128 per 100,000 people since 2010. However, this progress slightly reversed to 133 cases per 100,000 in 2022, underscoring the need for quality patient care, early diagnosis, effective treatment, as well as addressing drug-resistant TB [10]. Furthermore, the STOP TB Partnership has united governments, non-governmental organizations, the private sector, and affected communities to intensify efforts against TB [10].

Spatial analysis has been used as a valuable tool for understanding and addressing TB offering insights into its geographic distribution, clusters and risk factors [11]. By identifying hotspots and tracking patterns, spatial analysis enables targeted intervention in regions with high case concentrations [12, 13]. These clusters may arise from direct transmission or reactivation of latent TB among high-risk groups such as migrants [14]. Mapping TB cases supports more effective control measures by pinpointing outbreak sources and visualizing trends over time. Given TB unique characteristics such as its long latency and extended infectious period, spatial clusters may not always indicate active transmission but reflect high-risk populations mobility between regions [15, 16]. This study aimed to examine the geographical patterns of TB notifications in Nepal from the fiscal years (FY) 2020–2023 focusing on the environmental determinants of TB risk including land surface temperature during the day (LSTD) and night (LSTN), urbanization, precipitation, and cropland coverage.

## Methods

### Study area

Nepal, located in South Asia between India and China, spans 147,516 square kilometers and is characterized by diverse terrain, ranging from the low-lying tropical Terai region at 59 m to the towering Himalayan peaks, including Mount Everest at 8848 m. This geographical diversity presents significant challenges for public health efforts, including TB management. The country experiences a wide variation in climate, with temperatures ranging from tropical heat to freezing cold and annual precipitation levels from 160 mm in the northern Himalayan region to 5500 mm on the windward slopes. Nepal experiences diverse temperature ranges due to its varied topography. The coldest month is January with average minimum and maximum temperatures ranging from 4.6 to 18.1 °C. Conversely, June is the warmest month with temperatures typically spanning from 20.5 to 30.4 °C. Seasonal variation plays a significant role with cooler conditions in the mountainous regions and warmer temperatures in the lowland terai areas [17, 18].

**Fig. 1:**
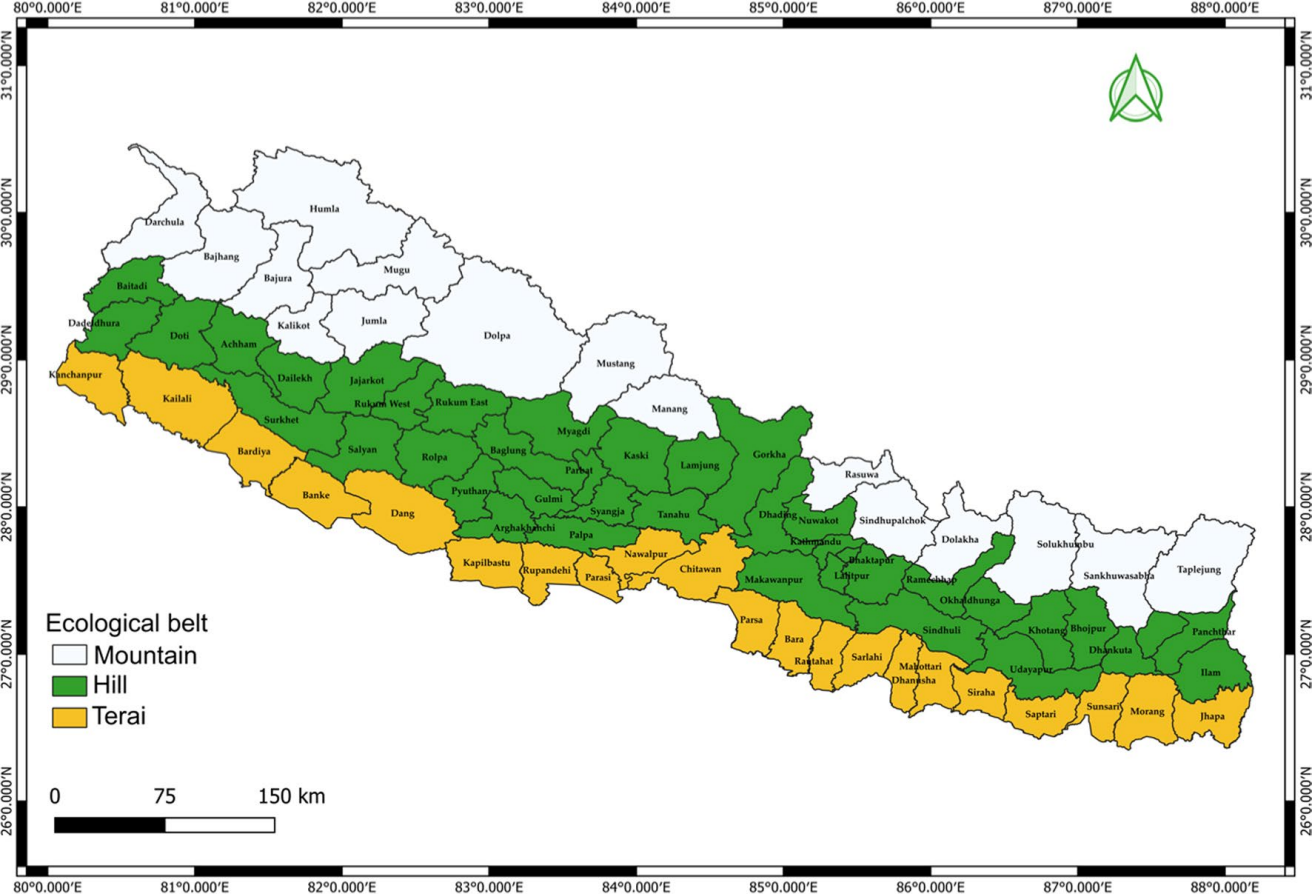
Ecological belt of Nepal

Administratively, Nepal is divided into seven provinces and 77 districts, further subdivided into 753 local levels, including metropolitan and rural municipalities. These divisions were used to map TB cases and environmental factors for spatial analysis. As of the National Population and Housing Census 2021, Nepal’s total population is approximately 29,164,578 [19]. The study utilized the Nepal Administrative Boundary [World Geodetic System 1984 (WGS 1984)] to obtain geographic coordinates for accurate spatial analysis, enabling an examination of how Nepal’s varied geography affects TB distribution and the environmental factors contributing to TB prevalence in FY 2020–2023. The study utilized fiscal years as the unit of analysis where each fiscal year spans from July 16 of one calendar year to July 15 of the following year (Fig. 1).

### Source of data variables

The data for this study were sourced from multiple platforms. Secondary environmental data were retrieved using the Google Earth Engine. Land Surface Temperature data for both day and night (pixel size: 1e13, spatial scale: 500 m) were acquired from the MOD09A1.061 dataset provided by NASA’s Land Processes Distributed Active Archive Center (LP DAAC) at the USGS EROS Center (https://developers.google.com/earth-engine/datasets/catalog/MODIS_061_MOD09A1). Datasets for cropland and urban areas (pixel size: 1e13, spatial scale: 100) were obtained from the Copernicus Global Land Cover Layers: CGLS-LC100 Collection 3, facilitated by The Copernicus Land Monitoring Service (https://developers.google.com/earth-engine/datasets/catalog/COPERNICUS_Landcover_100m_Proba-V-C3_Global). Precipitation data (pixel size: 1e13, spatial scale: 5566) were sourced from the Climate Hazards Group InfraRed Precipitation with Station data (CHIRPS), specifically the CHIRPS Pentad dataset (https://developers.google.com/earth-engine/datasets/catalog/UCSB-CHG_CHIRPS_PENTAD). The dependent variables were obtained from the National Tuberculosis Control Center Nepal for FY 2020–2023. The tuberculosis prevalence data available on an annual basis include, all diagnosed TB cases identified through pulmonary bacteriologically confirmed and pulmonary clinically diagnosed. These data cover a range of TB cases including pediatric TB, extra-pulmonary TB, TB-HIV co-infections and recurrent TB cases [9].

### Data preparation

We prepared, validated, and cleaned the raw dataset to guarantee accuracy and reliability. Once validation was complete, we imported the data into Quantum GIS (QGIS) version 3.40 [Open Source Geospatial Foundation (OSGeo), which is based in the United States] where spatial and non-spatial data were integrated into a shapefile for analysis (Nepal Administrative Boundary—WGS 1984) [20]. QGIS was used to visualize TB prevalence from FY 2020–2023 to detect patterns and identify potential clusters of high prevalence rates. Following this, we conducted an in-depth spatial analysis using GeoDa, version 1.22 (the Center for Spatial Data Science at the University of Chicago, Illinois, USA), a free, open-source tool developed by Dr. Luc Anselin which supports exploratory spatial data analysis including spatial autocorrelation (Moran’s *I*, Gi* and LISA) and regression through an intuitive interface [21].

### Data analysis

#### Gi statistics

The Getis-Ord Gi* statistic was employed in the data analysis to identify spatial clusters of TB prevalence across Nepal’s districts from fiscal year 2020 to 2023. This spatial analysis technique detected statistically significant hotspots (clusters of high TB cases) and cold spots (clusters of low TB cases) by comparing the observed TB cases in a district with those in neighboring districts. The Gi* statistic calculated whether the spatial concentration of TB cases in a given district and its neighbors was significantly higher or lower than the expected average [22].

Getis-Ord Gi* statistic was computed using the following formula.

1 $${\text{Gi}}*\, = \,\frac{{\sum {j\omega ij\chi j} }}{{\sum {j\chi j} }}$$

where the weights are determined by ω_ij_, and the normalization is done by dividing by the total sum of χ_j_ values.

A weight matrix was defined using the K-Nearest neighbors (K = 3) because of 3 ecological belt including mountains, hilly and terai regions. It considered neighboring relationships based on the three closest districts. Districts that exhibited significantly higher than expected prevalence of TB in relation to their neighbors were identified as hotspots while districts with lower-than-expected prevalence were labeled as cold spots. By applying the Gi* statistic, spatial patterns of TB transmission were uncovered, highlighting regions with persistently high or low disease prevalence and providing insights into where targeted public health interventions would have been most effective [23].

### Local indicators of spatial association (LISA)

Local Moran’s* I* was computed using the equation:

2 $$Local\,Moran\prime s\,I\, = \,\frac{{\left( {X_{i} \, - \,\overline{X}} \right)\,\sum {jW_{ij} \,\left( {X_{j} \, - \,\overline{X}} \right)} }}{{s_{i}^{2} }}$$

where "N" is the number of spatial units and "W*ij*" refers to the spatial weight between regions *i* and *j*.

Spatial autocorrelation for this study was evaluated using Local Indicators of Spatial Association, a set of statistics that includes measures like Moran’s *I* to detect various spatial patterns [24]. We applied LISA to assess the global spatial autocorrelation of TB prevalence and related factors. Specifically, Moran’s* I* was used within the LISA framework to identify whether particular regions formed part of spatial clusters with similar TB prevalence values (such as high-high or low-low clusters) or if they were outliers (like high-low or low–high clusters). This study used 999 permutations to assess the sensitivity of significant locations to the number of permutations with a significance threshold set at *P* < 0.05.

### Regression analysis

Multivariate analysis assesses the combined effects of multiple risk factors on tuberculosis cases while controlling for potential confounding variables, offering a more comprehensive understanding of the determinants of TB. We evaluated the *P-*values and Moran’s *I* value during the bivariate analysis of each variable. Variables with *P* < 0.05 were subsequently included in the regression model. Three types of multivariate analyses were employed in this study. The Ordinary Least Squares (OLS) model examines the relationship between TB cases and multiple independent variables, providing coefficients that quantify the influence of each risk factor. However, this method assumes no spatial dependence among the variables [25]. OLS regression should only be used if spatial autocorrelation is minimal or absent. Given the presence of significant positive spatial autocorrelation (as indicated by Moran’s *I*), reliance on OLS regression without addressing the spatial autocorrelation would be methodologically inappropriate. Therefore, OLS regression was only used when spatial autocorrelation was found to be minimal. If spatial autocorrelation was present, we addressed this issue through Spatial Error Models (SEM) and Spatial Lag Models (SLM).

The Spatial Error Model (SEM) addresses spatial autocorrelation in the error terms, a common issue in spatial data. By incorporating spatial dependencies, SEM enhances the model’s accuracy by ensuring that the error terms are not spatially correlated [26]. The Spatial Lag Model (SLM) incorporates a spatially lagged dependent variable to account for the influence of TB cases in neighbouring regions on a given area. This model effectively captures significant spatial dependencies, such as external effects and spatial interactions, manifested in the spatial lag *Wy* of the dependent variable Y [27]. We utilized a backward elimination regression approach, inserting variables into the model one at a time based on their significance in the bivariate analysis. If a variable did not demonstrate significance upon being added to the model, it was excluded from further consideration.

The weight matrix is crucial for determining the nature and extent of spatial interactions among the regions under investigation, aiding in the identification of spatial patterns like clusters or dispersions by defining neighboring regions and their influences on one another [28]. This study utilized a 3k-nearest neighbor weight matrix for both Gi*s statistics, univariate and bivariate analyses, and a Queen Contiguity weight matrix for spatial regression analysis. The 3k-nearest neighbor weight matrix identifies each location’s neighbors based on the three closest geographic units ensuring a consistent number of neighbors across regions. Conversely, the Queen Contiguity weight matrix considers regions as neighbors if they share a boundary or vertex, reflecting how a queen moves in chess. This method effectively captures the direct spatial dependencies and interactions among contiguous regions.

## Results

### TB clusters using Gi* statistics

**Fig. 2:**
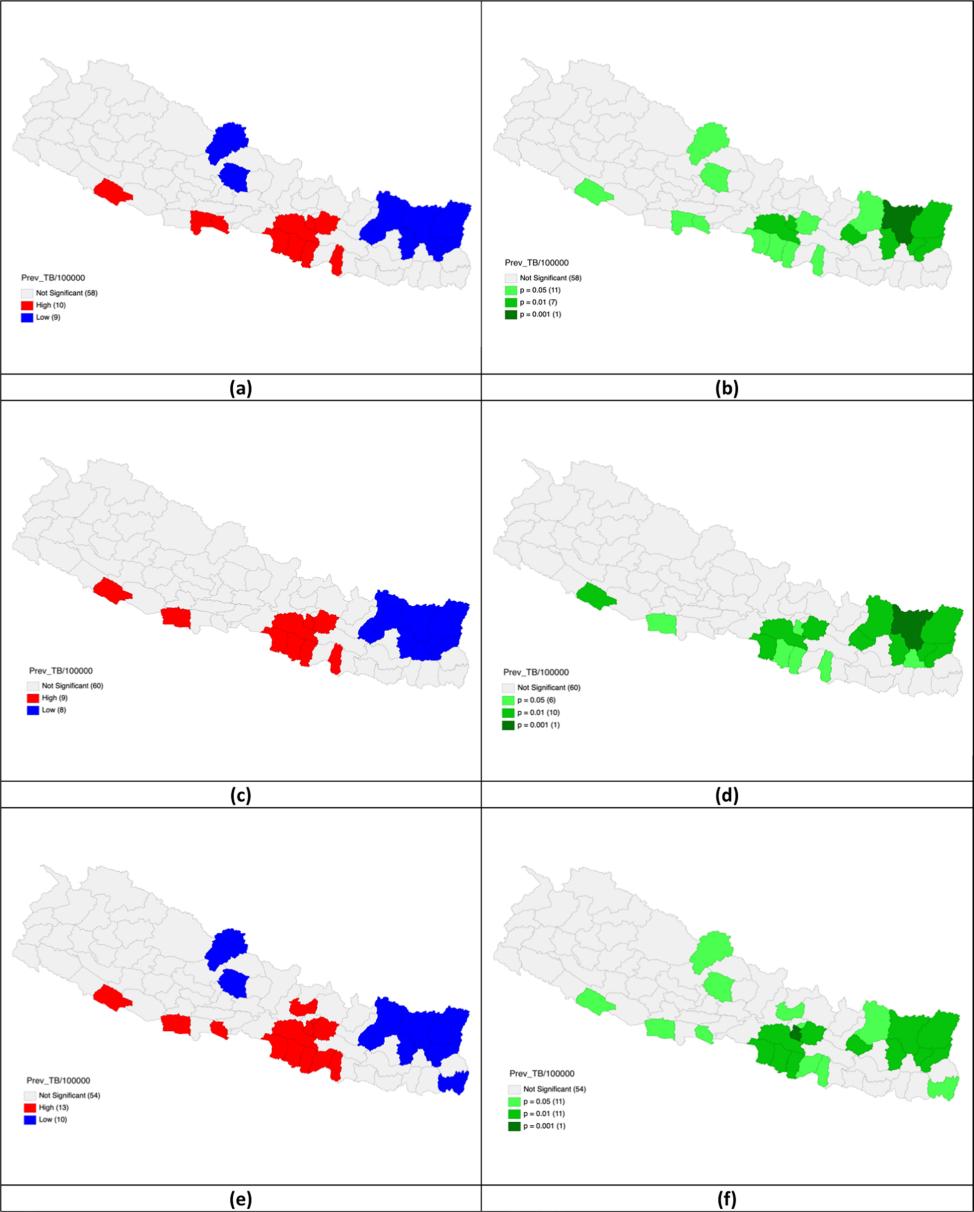
Univariate analysis of TB prevalence per 100,000 population in FY 2020–2023. **a** Gi* cluster map of TB prevalence per 100,000 population in FY 2020–2021; **b** Significant map of TB prevalence per 100,000 population in FY 2020–2021; **c** Gi* cluster map of TB prevalence per 100,000 population in FY 2021–2022; **d** Significant map of TB prevalence per 100,000 population in FY 2021–2022; **e** Gi* cluster map of TB prevalence per 100,000 population in FY 2022–2023; **f** Significant map of TB prevalence per 100,000 population in FY 2022–2023

In FY 2020–2021, the prevalence of TB per 100,000 population was high in the districts of Banke, Rupandehi, Parasi, Parsa, Bara, Rautahat, Mahottari, Makawanpur, Lalitpur, and Kabhrepalanchok, while it was low in Mustang, Kaski, Solukhumbu, Okhaldhunga, Bhojpur, Sankhuwasabha, Terhathum, Taplejung, and Panchthar. By FY 2021–2022, the Gi statistics indicated high TB prevalence in Banke, Kapilbastu, Parsa, Bara, Rautahat, Mahottari, Makawanpur, Lalitpur, and Kabhrepalanchok, and low prevalence in Solukhumbu, Okhaldhunga, Bhojpur, Sankhuwasabha, Terhathum, Taplejung, Panchthar, and Dhankuta. During FY 2022–2023, high TB rates were found in Banke, Kapilbastu, Parasi, Parsa, Bara, Rautahat, Sarlahi, Mahottari, Makawanpur, Lalitpur, Kabhrepalanchok, Bhaktapur, and Nuwakot, while low rates were observed in Mustang, Kaski, Solukhumbu, Okhaldhunga, Bhojpur, Sankhuwasabha, Terhathum, Taplejung, Panchthar, and Jhapa (Table 1, Fig. 2).

**Table 1:** Univariate analysis of TB prevalence per 100,000 population in the fiscal years 2020–2023

Fiscal year	Gi* statistics	
	High	Low
2020–2021	Banke* Rupandehi* Parasi* Parsa* Bara* Rautahat* Mahottari* Makawanpur** Lalitpur** Kabhrepalanchok*	Mustang* Kaski* Solukhumbu* Okhaldhunga** Bhojpur** Sankhuwasabha*** Terhathum** Taplejung** Panchthar**
2021–2022	Banke** Kapilbastu* Parsa** Bara* Rautahat* Mahottari* Makawanpur** Lalitpur* Kabhrepalanchok**	Solukhumbu** Okhaldhunga** Bhojpur** Sankhuwasabha*** Terhathum** Taplejung** Panchthar** Dhankuta*
2022–2023	Banke* Kapilbastu* Parasi* Parsa** Bara** Rautahat** Sarlahi* Mahottari* Makawanpur** Lalitpur*** Kabhrepalanchok** Bhaktapur* Nuwakot*	Mustang* Kaski* Solukhumbu* Okhaldhunga** Bhojpur** Sankhuwasabha** Terhathum** Taplejung** Panchthar** Jhapa*

Gi* Getis-Ord

*P* < 0.05*, *P* < 0.01**, *P* < 0.005***

### Univariate analysis of environmental factors

**Fig. 3:**
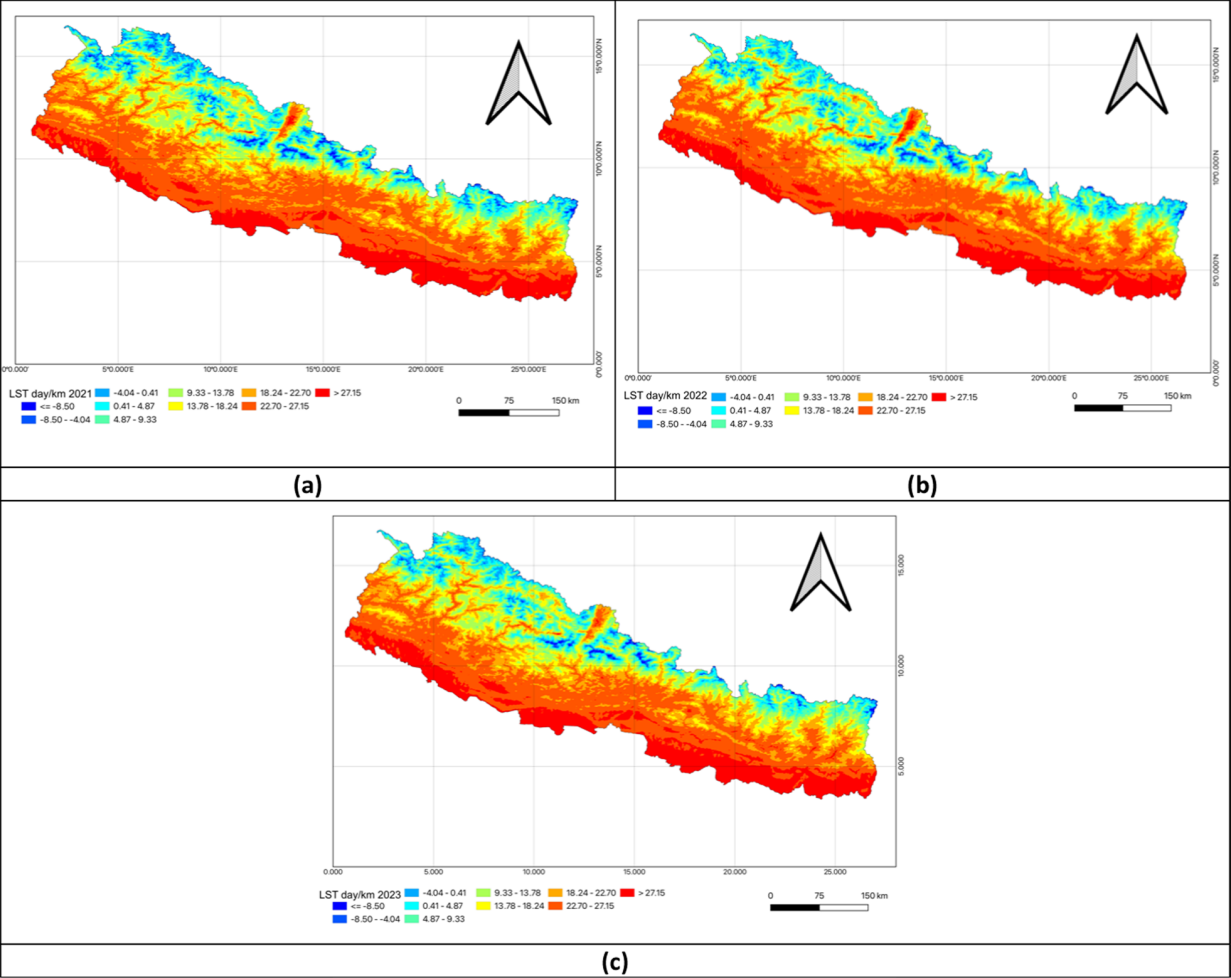
Univariate analysis of land surface temperature day in FY 2020–2023. **a** Land surface temperature day (LSTD) in FY 2020–2021; **b** Land surface temperature day (LSTD) in FY 2021–2022; **c** Land surface temperature day (LSTD) in FY 2022–2023

Figure 3 presents a visual analysis of Land Surface Temperature during the Day across Nepal. It highlighted the stark temperature differences influenced by elevation: the southern lowland Terai region experienced warmer daytime temperatures due to its lower altitude while the northern Himalayan regions remained cooler because of higher elevation and snow cover. The mid-hill regions showed moderate daytime temperatures, with variations based on altitude and terrain. These maps reflected the overall trend of warmer south and cooler north, illustrating Nepal’s diverse climate zones shaped by its topographical features in FY 2020–2023 (Fig. 3).

**Fig. 4:**
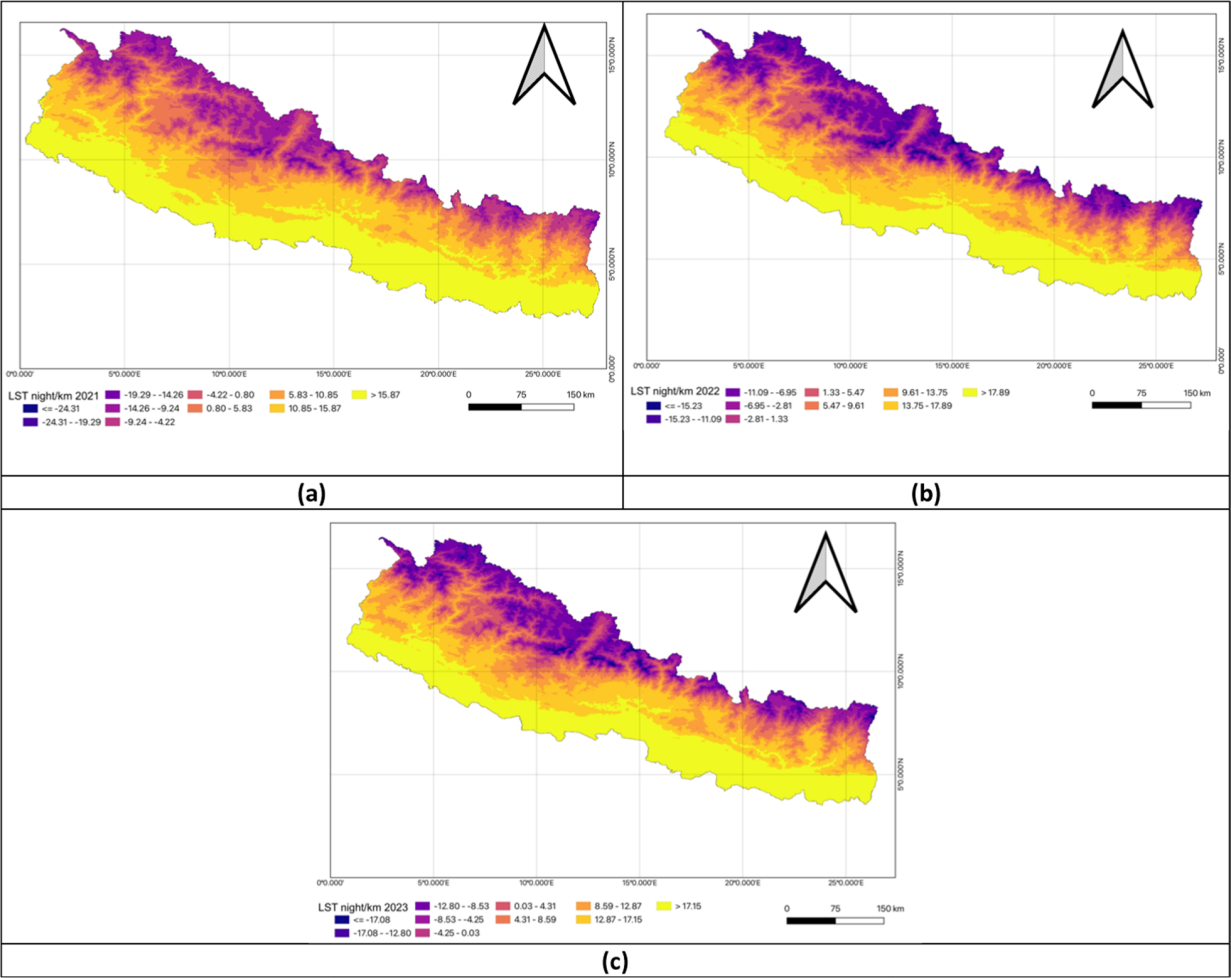
Univariate analysis of land surface temperature night in FY 2020–2023. **a** Land surface temperature night (LSTN) in FY 2020–2021; **b** Land surface temperature night (LSTN) in FY 2021–2022; **c** Land surface temperature night (LSTN) in FY 2022–2023

Figure 4 provided a visual representation of Land Surface Temperature at Night across Nepal. The southern plains consistently experience warmer nighttime temperatures while the northern mountainous regions remain cooler. These maps could reflect different time periods, models, or averages for LSTN but the overall pattern of warmer south and cooler north remains consistent (Fig. 4).

**Fig. 5:**
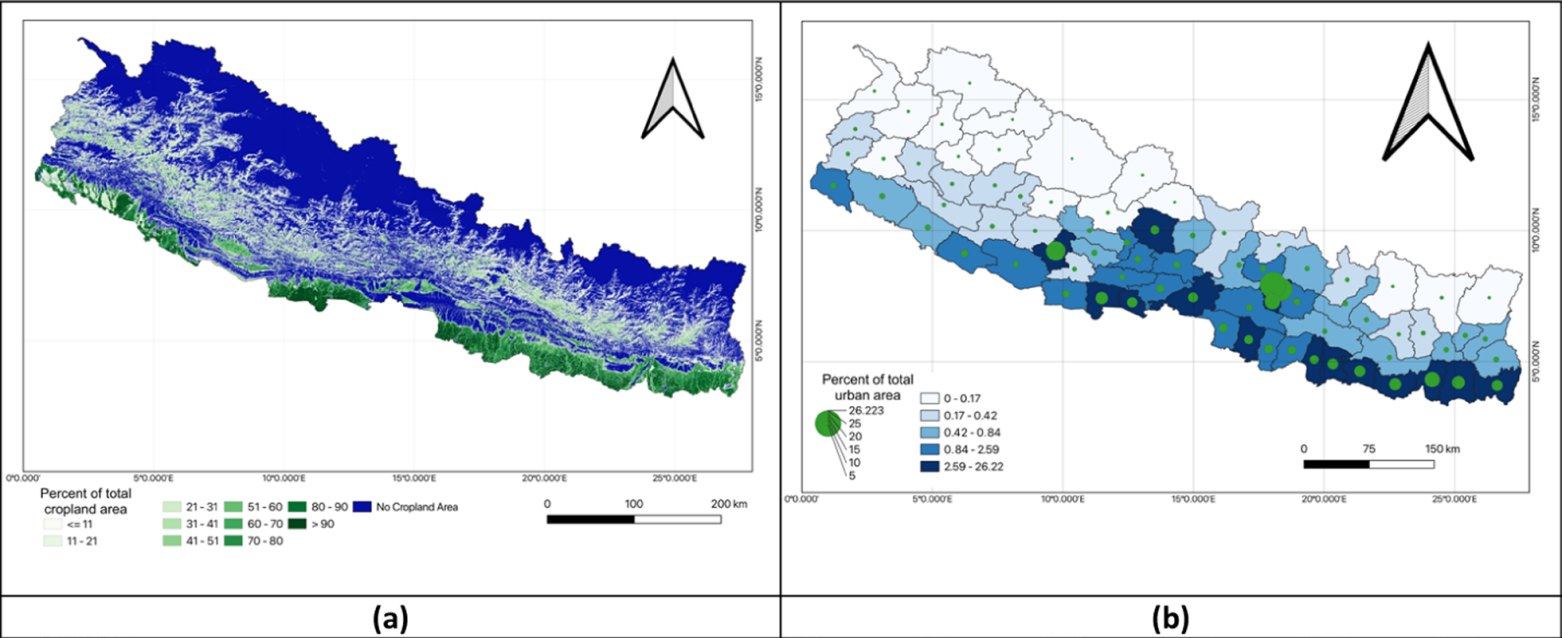
Univariate analysis of percent of total cropland area and percent of total urban area. **a** Percent of total cropland area; **b** Percent of total urban area

Figure 5 of the left map illustrates the distribution of cropland across Nepal showing the percent of the total cropland area. The different color shades, ranging from green to blue, represented varying percentages with the green areas concentrated primarily in the southern Terai region, indicating a higher proportion of cropland. In contrast, the northern regions which were more mountainous appear in blue reflecting a lower percentage of cropland. The right map focused on the percent of the total urban area across different districts. Shades of blue represented the degree of urbanization with darker shades indicating districts with a higher percentage of urban land. Additionally, green circles of varying sizes mark locations where urbanization was more prominent with larger circles corresponding to areas of greater urban concentration. Urban areas were predominantly located in the southern and central parts of the country while the northern mountainous regions showed minimal urban development (Fig. 5).

**Fig. 6:**
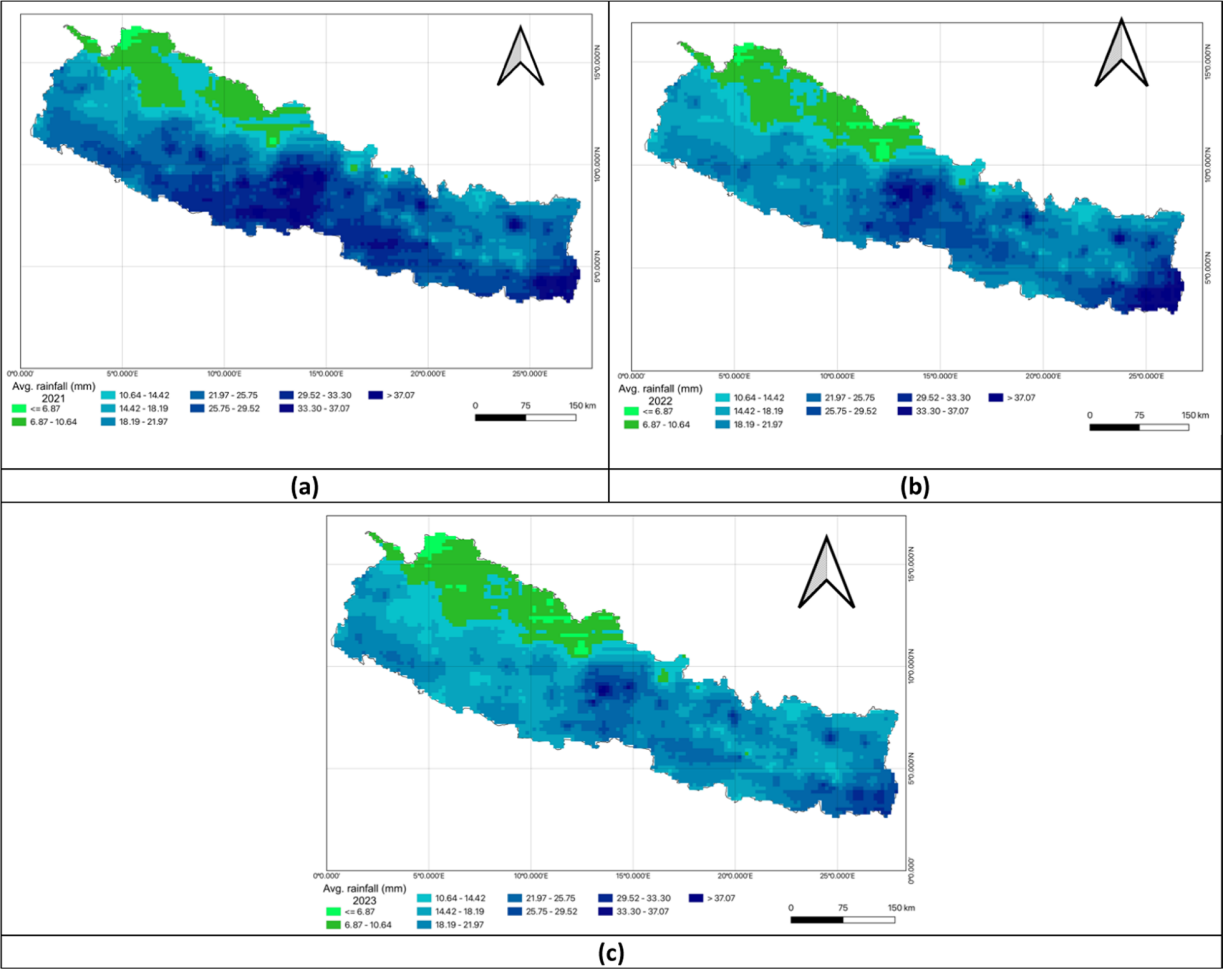
Univariate analysis of rainfall in FY 2020–2023. **a** Rainfall in FY 2020–2021; **b** Rainfall in FY 2021–2022 **c** Rainfall in FY 2022–2023

Figure 6 provides a visual comparison of rainfall patterns in Nepal for the fiscal years 2020–2023. While northern Nepal consistently experienced lower rainfall, central and southern regions saw higher precipitation levels, especially in FY 2022–2023 where rainfall intensified. The darker shade in the map suggested a cumulative or average representation of rainfall over these years highlighting regions that received the most rainfall (Fig. 6).

### Bivariate analysis of the prevalence of tuberculosis in FY 2020–2023

#### Land surface temperature day (LSTD) and tuberculosis prevalence

**Fig. 7:**
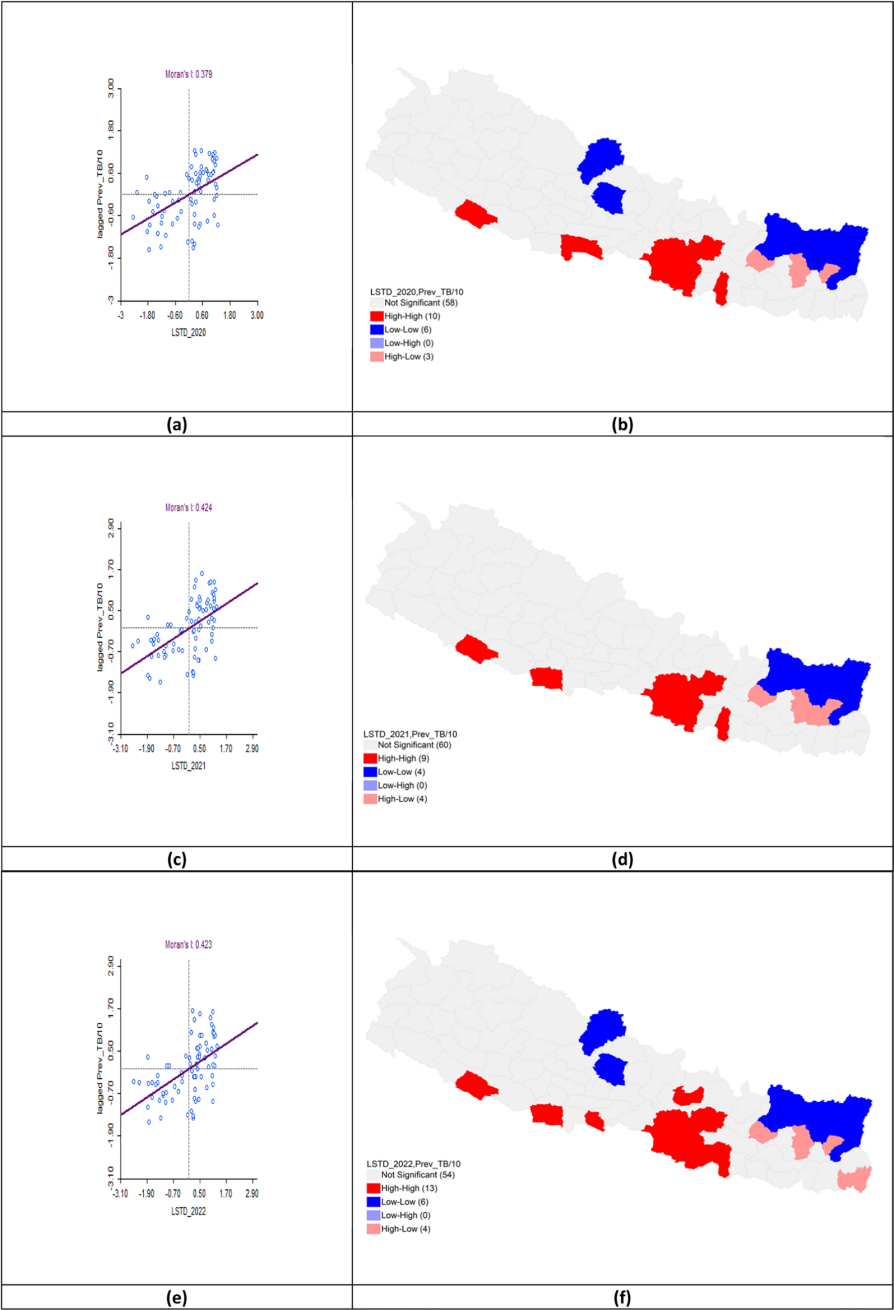
Bivariate analysis of land surface temperature day and TB prevalence per 100,000 population in FY 2020–2023. **a** Moran’s *I* scatter plot of land surface temperature day and TB prevalence per 100,000 population in 2020–2021; **b** LISA map of land surface temperature day and TB prevalence per 100,000 population in 2020–2021; **c** Moran’s *I* scatter plot of land surface temperature day and TB prevalence per 100,000 population in 2021–2022; **d** LISA map of land surface temperature day and TB prevalence per 100,000 population in 2021–2022; **e** Moran’s* I* scatter plot of land surface temperature day and TB prevalence per 100,000 population in 2022–2023; **f** LISA map of land surface temperature day and TB prevalence per 100,000 population in 2022–2023

The bivariate LISA analysis revealed a statistically significant positive autocorrelation between LSTD and TB prevalence across all three fiscal years. In FY 2020–2021, Moran’s *I* was 0.379. High-High clusters were identified in Banke, Rupandehi, Parasi, Parsa, Bara, Rautahat, Makawanpur, Lalitpur, Kabhrepalanchok, and Mahottari, while Low-Low clusters appeared in Mustang, Kaski, Solukhumbu, Sankhuwasabha, Taplejung, and Panchthar. In FY 2021–2022, Moran’s *I* increased to 0.424, with hotspots persisting in Banke, Kapilbastu, Parsa, Bara, Rautahat, Makawanpur, Lalitpur, Kabhrepalanchok, and Mahottari. Cold spots remained in Solukhumbu, Sankhuwasabha, Taplejung, and Panchthar. For FY 2022–2023, Moran’s *I* was 0.423, with hotspot clusters expanding to include Sarlahi, Bhaktapur, and Nuwakot. Cold spots persisted in Mustang, Kaski, Solukhumbu, Sankhuwasabha, Taplejung, and Panchthar (Table 2, Fig. 7).

**Table 2:** Bivariate analysis of land surface temperature day and TB prevalence per 100,000 population in the fiscal years 2020–2023

Fiscal year	LISA				Moran’s *I*
	HH	LL	HL	LH	
2020–2021	Banke* Rupandehi* Parasi* Parsa* Bara* Rautahat* Makawanpur** Lalitpur** Kabhrepalanchok* Mahottari*	Mustang* Kaski* Solukhumbu* Sankhuwasabha*** Taplejung** Panchthar**	Okhaldhunga** Bhojpur** Terhathum**		0.379
2021–2022	Banke** Kapilbastu* Parsa** Bara* Rautahat* Makawanpur** Lalitpur* Kabhrepalanchok** Mahottari*	Solukhumbu** Sankhuwasabha*** Taplejung** Panchthar**	Okhaldhunga** Bhojpur** Terhathum** Dhankuta*		0.424
2022–2023	Banke* Kapilbastu* Parasi* Parsa** Bara** Rautahat** Sarlahi* Mahottari* Makawanpur** Lalitpur*** Kabhrepalanchok** Bhaktapur* Nuwakot*	Mustang* Kaski* Solukhumbu* Sankhuwasabha** Taplejung** Panchthar**	Okhaldhunga** Bhojpur** Terhathum** Jhapa*		0.423

*LISA* Local indicators of spatial associations, *HH* High-High, *LL* Low-Low, *HL* High-Low, *LH* Low–High

*P* < 0.05*, *P* < 0.01**, *P* < 0.005***

#### Land surface temperature night and tuberculosis prevalence

**Fig. 8:**
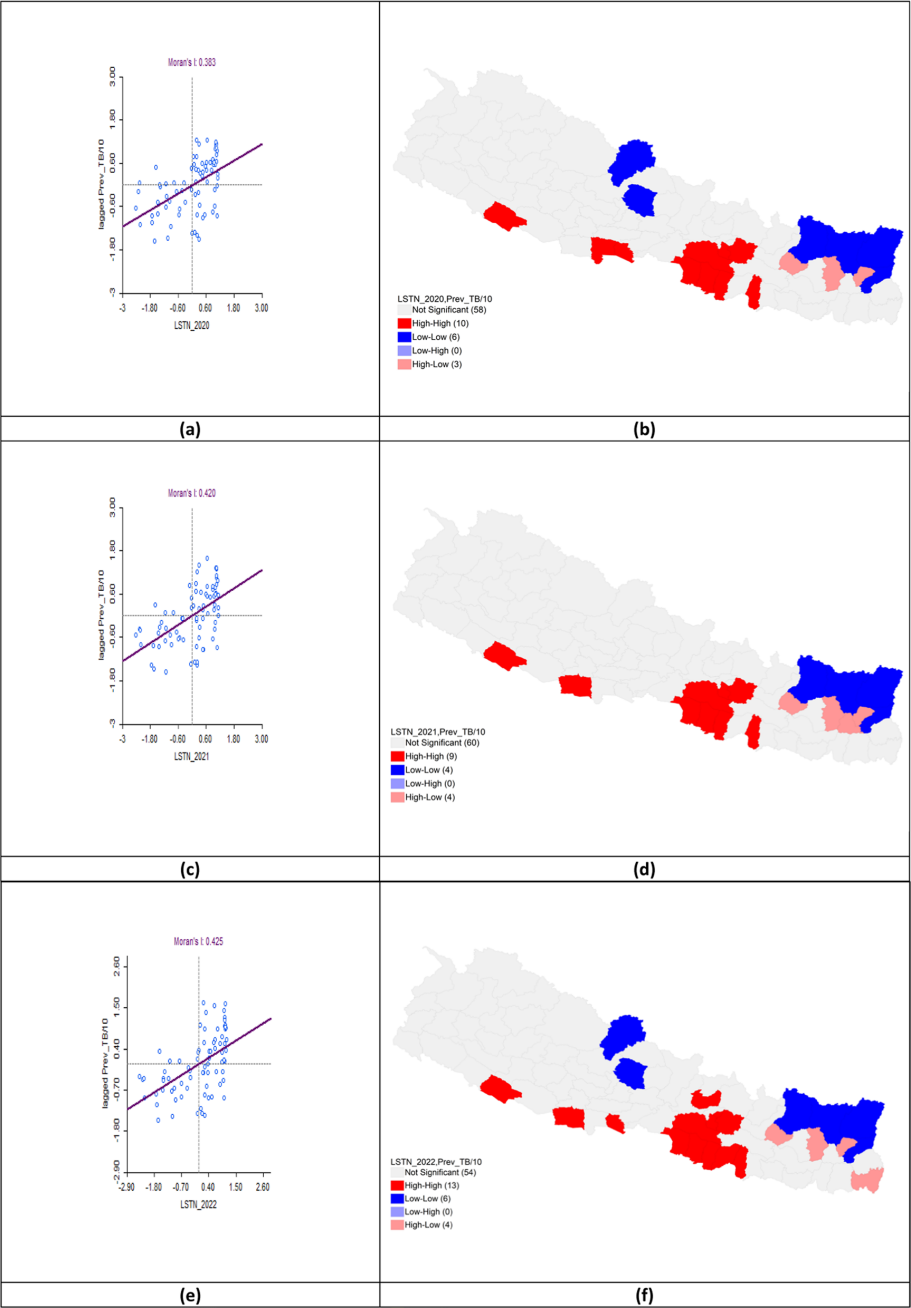
Bivariate analysis of land surface temperature night and TB prevalence per 100,000 population in FY 2020–2023. **a** Moran’s *I* scatter plot of land surface temperature night and TB prevalence per 100,000 population in 2020–2021; **b** LISA map of land surface temperature night and TB prevalence per 100,000 population in 2020–2021; **c** Moran’s *I* scatter plot of land surface temperature night and TB prevalence per 100,000 population in 2021–2022; **d** LISA map of land surface temperature night and TB prevalence per 100,000 population in 2021–2022; **e** Moran’s *I* scatter plot of land surface temperature night and TB prevalence per 100,000 population in 2022–2023; **f** LISA map of land surface temperature night and TB prevalence per 100,000 population in 2022–2023

The bivariate LISA analysis showed a statistically significant positive correlation between LSTN and TB prevalence across all three fiscal years. In FY 2020–2021, Moran’s *I* was 0.383. High-High clusters were identifie in Banke, Rupandehi, Parasi, Parsa, Bara, Rautahat, Makawanpur, Lalitpur, Kabhrepalanchok, and Mahottari, while Low-Low clusters appeared in Mustang, Kaski, Solukhumbu, Sankhuwasabha, Taplejung and Panchthar. In FY 2021–2022, Moran’s* I* increased to 0.420 with hotspots persisting in Banke, Kapilbastu, Parsa, Bara, Rautahat, Makawanpur, Lalitpur, Kabhrepalanchok, and Mahottari, while cold spots remained in Solukhumbu, Sankhuwasabha, Taplejung and Panchthar. For FY 2022–2023, Moran’s *I* rose to 0.425. Hotspots expanded to include Sarlahi, Bhaktapur, and Nuwakot, while cold-spot clusters persisted in Mustang, Kaski, Solukhumbu, Sankhuwasabha, Taplejung, and Panchthar (Table 3, Fig. 8).

**Table 3:** Bivariate analysis of land surface temperature night and TB prevalence per 100,000 population in the fiscal years 2020–2023

Fiscal year	LISA				Moran’s *I*
	HH	LL	HL	LH	
2020–2021	Banke* Rupandehi* Parasi* Parsa* Bara* Rautahat* Makawanpur** Lalitpur** Kabhrepalanchok* Mahottari*	Mustang* Kaski* Solukhumbu* Sankhuwasabha*** Taplejung** Panchthar**	Okhaldhunga** Bhojpur** Terhathum**		0.383
2021–2022	Banke** Kapilbastu* Parsa** Bara* Rautahat* Makawanpur** Lalitpur* Kabhrepalanchok** Mahottari*	Solukhumbu** Sankhuwasabha*** Taplejung** Panchthar**	Okhaldhunga** Bhojpur** Terhathum** Dhankuta*		0.420
2022–2023	Banke* Kapilbastu* Parasi* Parsa** Bara** Rautahat** Sarlahi* Mahottari* Makawanpur** Lalitpur*** Kabhrepalanchok** Bhaktapur* Nuwakot*	Mustang* Kaski* Solukhumbu* Sankhuwasabha** Taplejung** Panchthar**	Okhaldhunga** Bhojpur** Terhathum** Jhapa*		0.425

*LISA* Local indicators of spatial associations, *HH* High-High, *LL* Low-Low, *HL* High-Low, *LH* Low–High

*P* < 0.05*, *P* < 0.01**, *P* < 0.005***

### Cropland distribution and tuberculosis prevalence

**Fig. 9:**
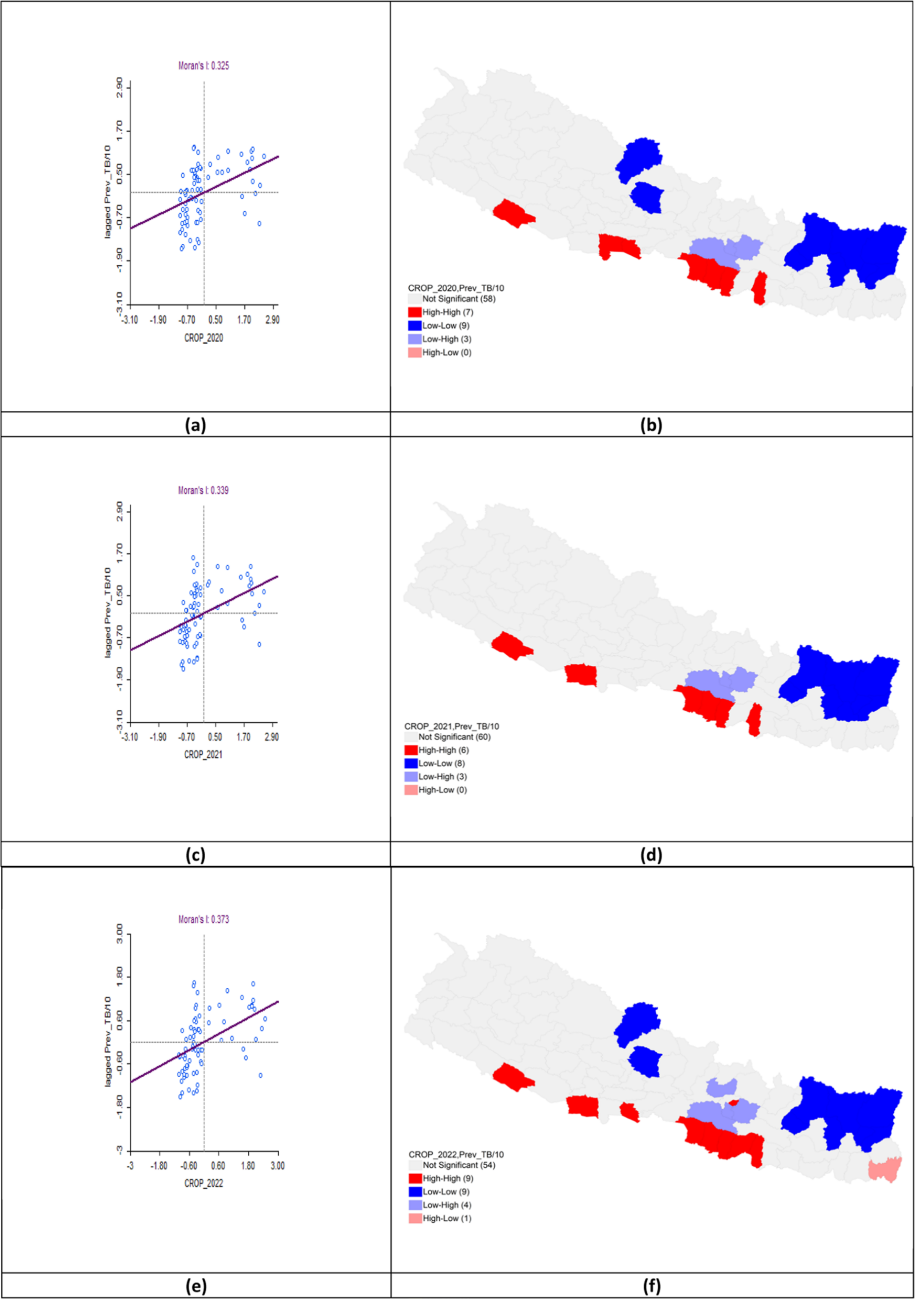
Bivariate analysis of cropland area and TB prevalence per 100,000 population in FY 2020–2023. **a** Moran’s *I* scatter plot of cropland area and TB prevalence per 100,000 population in 2020–2021; **b** LISA map of cropland area and TB prevalence per 100,000 population in 2020–2021; **c** Moran’s *I* scatter plot of cropland area and TB prevalence per 100,000 population in 2021–2022; **d** LISA map of cropland area and TB prevalence per 100,000 population in 2021–2022; **e** Moran’s *I* scatter plot of cropland area and TB prevalence per 100,000 population in 2022–2023; **f** LISA map of cropland area and TB prevalence per 100,000 population in 2022–2023

The bivariate LISA analysis demonstrated a statistically significant positive correlation between cropland area and TB prevalence across all three fiscal years. In FY 2020–2021, Moran’s *I* was 0.325 with High-High clusters in Banke, Rupandehi, Parasi, Parsa, Bara, Rautahat, and Mahottari, while Low-Low clusters appeared in Mustang, Kaski, Solukhumbu, Sankhuwasabha, Taplejung, Okhaldhunga, Bhojpur, Terhathum, and Panchthar. In FY 2021–2022, Moran’s *I* increased to 0.339 with hotspots persisting in Banke, Kapilbastu, Parsa, Bara, Rautahat and Mahottari while cold spots remained in Solukhumbu, Sankhuwasabha, Taplejung, Okhaldhunga, Bhojpur, Terhathum, Dhankuta, and Panchthar. For FY 2022–2023, Moran’s* I* rose to 0.373 with hotspots expanding to include Sarlahi and Bhaktapur. Cold-spot clusters persisted in Mustang, Kaski, Solukhumbu, Sankhuwasabha, Taplejung, Okhaldhunga, Bhojpur, Terhathum, and Panchthar (Table 4, Fig. 9).

**Table 4:** Bivariate analysis of Cropland area and TB prevalence per 100,000 population in the fiscal years 2020–2023

Fiscal year	LISA				Moran’s *I*
	HH	LL	HL	LH	
2020–2021	Banke* Rupandehi* Parasi* Parsa* Bara* Rautahat* Mahottari*	Mustang* Kaski* Solukhumbu* Sankhuwa sabha*** Taplejung** Panchthar** Okhaldhunga** Bhojpur** Terhathum**		Makawanpur** Lalitpur** Kabhrepalan chok*	0.325
2021–2022	Banke** Kapilbastu* Parsa** Bara* Rautahat* Mahottari*	Solukhumbu** Sankhuwa sabha*** Taplejung** Panchthar** Okhaldhunga** Bhojpur** Terhathum** Dhankuta*		Makawanpur** Lalitpur* Kabhrepalan chok**	0.339
2022–2023	Banke* Kapilbastu* Parasi* Parsa** Bara** Rautahat** Sarlahi* Mahottari* Bhaktapur*	Mustang* Kaski* Solukhumbu* Sankhuwa sabha** Taplejung** Panchthar** Okhaldhunga** Bhojpur** Terhathum**	Jhapa*	Makawanpur** Lalitpur*** Kabhrepalan chok** Nuwakot*	0.373

*LISA* Local indicators of spatial associations, *HH* High-High, *LL* Low-Low, *HL* High-Low, *LH* Low–High

*P* < 0.05*, *P* < 0.01**, *P* < 0.005***

### Urbanization and tuberculosis prevalence

**Fig. 10:**
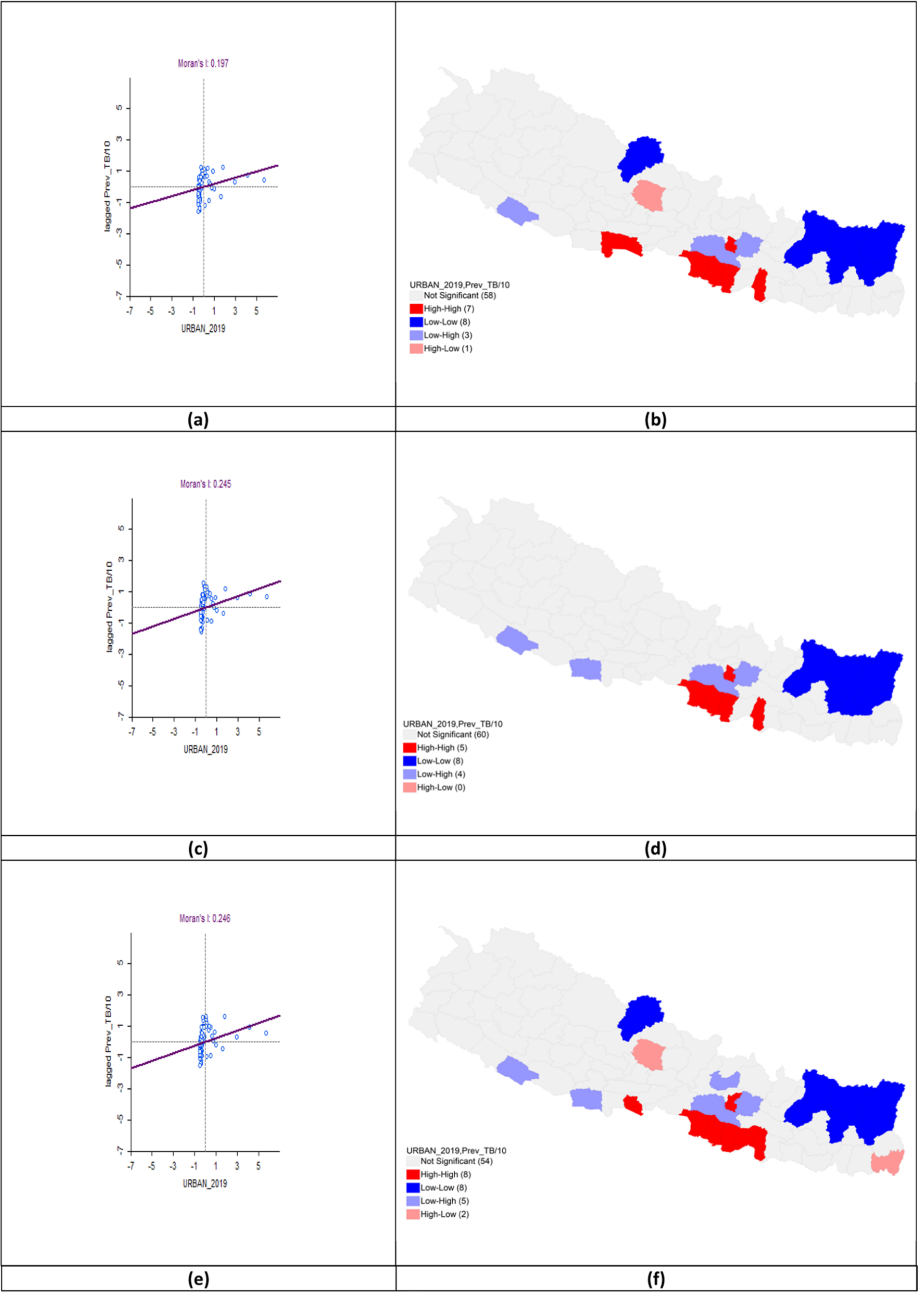
Bivariate analysis of urbanization and TB prevalence per 100,000 population in FY 2020–2023. **a** Moran’s *I* scatter plot of urbanization and TB prevalence per 100,000 population in 2020–2021; **b** LISA map of urbanization and TB prevalence per 100,000 population in 2020–2021; **c** Moran’s *I* scatter plot of urbanization and TB prevalence per 100,000 population in 2021–2022; **d** LISA map of urbanization and TB prevalence per 100,000 population in 2021–2022; **e** Moran’s *I* scatter plot of urbanization and TB prevalence per 100,000 population in 2022–2023; **f** LISA map of urbanization and TB prevalence per 100,000 population in 2022–2023

The bivariate LISA analysis revealed a statistically significant positive correlation between urbanization and TB prevalence across all three fiscal years. In FY 2020–2021, Moran’s *I* was 0.197 with High-High clusters in Rupandehi, Parasi, Parsa, Bara, Rautahat, Lalitpur, and Mahottari, while Low-Low clusters appeared in Mustang, Solukhumbu, Sankhuwasabha, Taplejung, Okhaldhunga, Bhojpur, Terhathum, and Panchthar. In FY 2021–2022, Moran’s *I* increased to 0.245 with hotspots in Parsa, Bara, Rautahat, and Mahottari while cold spots remained in Solukhumbu, Sankhuwasabha, Taplejung, Okhaldhunga, Bhojpur, Terhathum, Dhankuta, and Panchthar. For FY 2022–2023, Moran’s *I* rose to 0.246 with hotspots expanding to include Parasi, Sarlahi, Lalitpur and Bhaktapur. Cold-spot clusters persisted in Mustang, Solukhumbu, Sankhuwasabha, Taplejung, Okhaldhunga, Bhojpur, Terhathum, and Panchthar (Table 5, Fig. 10).

**Table 5:** Bivariate analysis of Urbanization and TB prevalence per 100,000 population in the fiscal years 2020–2023

Fiscal year	LISA				Moran’s *I*
	HH	LL	HL	LH	
2020–2021	Rupandehi* Parasi* Parsa* Bara* Rautahat* Mahottari* Lalitpur**	Mustang* Solukhumbu* Sankhuwa sabha*** Taplejung** Panchthar** Okhaldhunga** Bhojpur** Terhathum**	Kaski*	Makawanpur** Kabhrepalan chok* Banke*	0.197
2021–2022	Parsa** Bara* Rautahat* Mahottari* Lalitpur*	Solukhumbu** Sankhuwa sabha*** Taplejung** Panchthar** Okhaldhunga** Bhojpur** Terhathum** Dhankuta*		Makawanpur** Kabhrepalan chok** Banke** Kapilbastu*	0.245
2022–2023	Parasi* Parsa** Bara** Rautahat** Sarlahi* Mahottari* Bhaktapur* Lalitpur***	Mustang* Solukhumbu* Sankhuwa sabha** Taplejung** Panchthar** Okhaldhunga** Bhojpur** Terhathum**	Jhapa* Kaski*	Makawanpur** Kabhrepalan chok** Nuwakot* Banke* Kapilbastu*	0.246

*LISA* Local indicators of spatial associations, *HH* High-High, *LL* Low-Low, *HL* High-Low, *LH* Low–High

*P* < 0.05*, *P* < 0.01**, *P* < 0.005***

### Precipitation patterns and tuberculosis prevalence

**Fig. 11:**
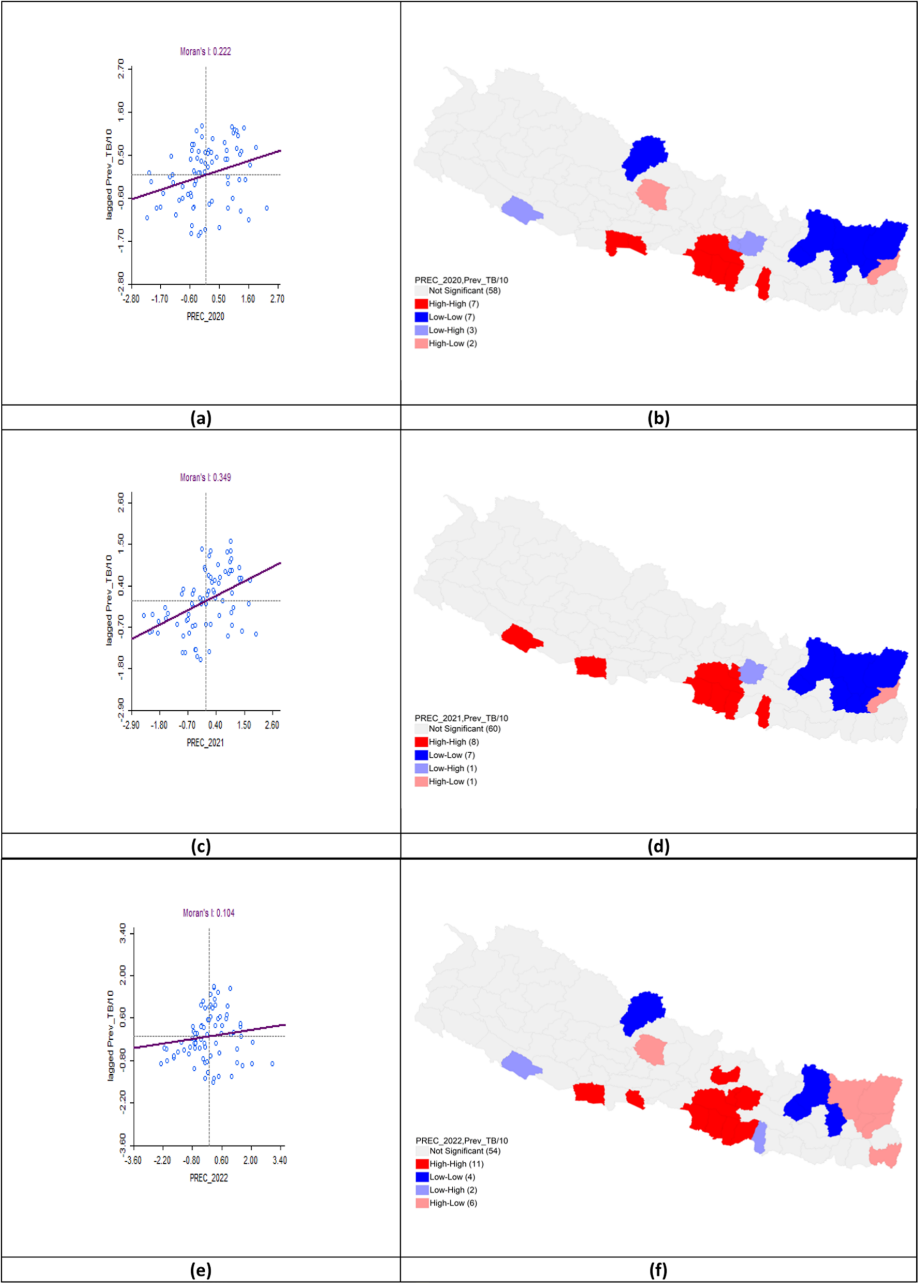
Bivariate analysis of precipitation and TB prevalence per 100,000 population in FY 2020–2023. **a** Moran’s *I* scatter plot of precipitation and TB prevalence per 100,000 population in 2020–2021; **b** LISA map of precipitation and TB prevalence per 100,000 population in 2020–2021; **c** Moran’s *I* scatter plot of precipitation and TB prevalence per 100,000 population in 2021–2022; **d** LISA map of precipitation and TB prevalence per 100,000 population in 2021–2022; **e** Moran’s *I* scatter plot of precipitation and TB prevalence per 100,000 population in 2022–2023; **f** LISA map of precipitation and TB prevalence per 100,000 population in 2022–2023

The bivariate LISA analysis revealed a statistically significant positive correlation between precipitation levels and TB prevalence across all three fiscal years. In FY 2020–2021, Moran’s* I* was 0.222 with High-High clusters in Rupandehi, Parasi, Parsa, Bara, Rautahat, Makawanpur, and Mahottari while Low-Low clusters appeared in Mustang, Solukhumbu, Sankhuwasabha, Taplejung, Okhaldhunga, Bhojpur, and Terhathum. In FY 2021–2022, Moran’s *I* increased to 0.349 with hotspots identified in Parsa, Bara, Rautahat, Lalitpur, Banke, Kapilbastu, Makawanpur, and Mahottari. Cold spots remained in Solukhumbu, Sankhuwasabha, Taplejung, Okhaldhunga, Bhojpur, Terhathum, Dhankuta, and Panchthar. For FY 2022–2023, Moran’s *I* decreased to 0.104 but the positive spatial autocorrelation persisted. Hotspot clusters expanded to include Parasi, Parsa, Bara, Rautahat, Sarlahi, Makawanpur, Kabhrepalanchok, Nuwakot, Kapilbastu, Lalitpur, and Bhaktapur. Cold-spot clusters characterized by low precipitation and low TB prevalence persisted in Mustang, Solukhumbu, Okhaldhunga, and Bhojpur (Table 6, Fig. 11).

**Table 6:** Bivariate analysis of Precipitation and TB prevalence per 100,000 population in the fiscal years 2020–2023

Fiscal year	LISA				Moran’s *I*
	HH	LL	HL	LH	
2020–2021	Rupandehi* Parasi* Parsa* Bara* Rautahat* Mahottari* Makawanpur**	Mustang* Solukhumbu* Sankhuwa sabha*** Taplejung** Okhaldhunga** Bhojpur** Terhathum**	Kaski* Panchthar**	Kabhrepalan chok* Banke* Lalitpur**	0.222
2021–2022	Parsa** Bara* Rautahat* Mahottari* Lalitpur* Banke** Kapilbastu* Makawanpur**	Solukhumbu** Sankhuwa sabha*** Taplejung** Okhaldhunga** Bhojpur** Terhathum** Dhankuta*	Panchthar**	Kabhrepalan chok**	0.349
2022–2023	Parasi* Parsa** Bara** Rautahat** Sarlahi* Bhaktapur* Lalitpur*** Makawanpur** Kabhrepalan chok** Nuwakot* Kapilbastu*	Mustang* Solukhumbu* Okhaldhunga** Bhojpur**	Jhapa* Kaski* Sankhuwa sabha** Taplejung** Panchthar** Terhathum**	Banke* Mahottari*	0.104

*LISA* Local indicators of spatial associations, *HH* High-High, *LL* Low-Low, *HL* High-Low, *LH* Low–High

*P* < 0.05*, *P* < 0.01**, *P* < 0.005***

### Regression analysis

The spatial modeling results from the OLS regression indicated that LSTN and urbanization were significant factors explaining approximately 40% of the variation in TB prevalence during FY 2020–2021 (R^2^ = 0.40). The SEM and SLM models further examined the influence of LSTN and urbanization revealing a strong relationship with TB prevalence in Nepal for the same period. The SEM model explained 65.5% of the variance (R^2^ = 0.655) while the SLM model accounted for 64% (R^2^ = 0.640). Of these, the SEM model proved to be the most effective. Parameter estimates from the SEM model showed that both LSTN (Coefficient: 1.534) and urbanization (Coefficient: 2.605) were positively autocorrelated with TB prevalence in 2020–2021. The R^2^ value demonstrated the SEM model’s ability to explain 65.5% of the variation in TB prevalence. Additionally, based on the AIC test, the SEM model slightly outperformed the SLM with AIC values of 695.49 and 697.326, respectively. Therefore, the SEM model was considered the most effective in explaining the spatial distribution of TB prevalence in Nepal for FY 2020–2021.

For FY 2021–2022, the OLS regression results showed that LSTN and urbanization remained significant accounting for 42.6% of the variation in TB prevalence (R^2^ = 0.426). The SEM and SLM models again identified a strong relationship between these factors and TB prevalence. The SEM model explained 71.2% of the variance (R^2^ = 0.712), while the SLM model explained 72.1% (R^2^ = 0.721). In this case, the SLM model emerged as the most effective. The parameter estimates from the SLM model indicated that LSTN (Coefficient: 1.206) and urbanization (Coefficient: 2.624) continued to show positive autocorrelation with TB prevalence in 2021–2022. The SLM model’s R^2^ value highlighted its ability to explain 72.1% of the variation in TB prevalence. Furthermore, the AIC test favored the SLM model over the SEM with AIC values of 731.009 and 732.964, respectively. Thus, the SLM model was determined to be more effective in explaining the spatial distribution of TB prevalence in Nepal for FY 2021–2022.

In FY 2022–2023, OLS regression results demonstrated that LSTN, precipitation, and urbanization were significant factors, accounting for 49.9% of the variation in TB prevalence (R^2^ = 0.499). The SEM and SLM models were again used to evaluate the impact of LSTN and urbanization confirming a strong association with TB prevalence during this period. The SEM model explained 68.9% of the variance (R^2^ = 0.689) while the SLM model accounted for 69.6% (R^2^ = 0.696). During this period, the SLM model was again found to be the most effective. The parameter estimates from the SLM model indicated a positive autocorrelation between LSTN (Coefficient: 1.750), urbanization (Coefficient: 2.643) and TB prevalence in 2022–2023. The SLM model’s R^2^ value captured 69.6% of the variation in TB prevalence. Additionally, based on the AIC test, the SLM model performed slightly better than the SEM, with AIC values of 724.45 and 726.924, respectively. Therefore, the SLM model was concluded to be more effective in explaining the spatial distribution of TB prevalence in Nepal for FY 2022–2023 (Table 7).

**Table 7:** Regression analysis for the prevalence of TB per 100,000 population in the fiscal years 2020–2023

Factors	2020–2021			2021–2022			2022–2023		
	OLS	SLM	SEM	OLS	SLM	SEM	OLS	SLM	SEM
LSTN	2.23***	1.09***	1.534***	3.046***	1.206**	1.607*	3.653***	1.750***	1.835**
Urban	2.13**	1.84***	2.605 ***	3.264**	2.624***	3.425***	3.456***	2.643***	3.168***
Precipitation							−0.366**	−**0.167**	0.012
Constant	49.24	12.52	**56.362**	62.618	**9.143**	77.142	96.256	**33.135**	71.413
ρ	–	0.64	–	–	**0.728**	–	–	**0.619**	–
λ (varies between 0.1 and 1.0)	–	–	**0.682**	–	–	0.760	–	–	0.701
F-stat (minimum value = 0)	26.36	–	–	29.221	–	–	26.196	–	–
R-Squared (coefficient of determination)	0.400	0.640	**0.655**	0.426	**0.721**	0.712	0.499	**0.696**	0.689
Log likelihood	−360.064	−344.816	−**344.745**	−382.381	−**361.505**	−363.482	−371.041	−**357.225**	−359.462
AIC	726.128	697.632	**695.49**	770.762	**731.009**	732.964	750.082	**724.45**	726.924
BIC	733.16	707.007	**702.522**	777.793	**740.384**	739.995	759.457	**736.169**	736.299

Bold values indicate the best regression model to predict the spatial association

*ρ* Rho,* λ* Lumda, *OLS* Oridnary least square, *SEM* Spatial error model, *SLM* Spatial lag model, *AIC* Akaike information crierion, *BIC* Bayesian information criterion, *F-stat* F-statistic

Coefficient significant at *0.05, **0.01, ***0.005

## Discussion

The analysis of TB prevalence in Nepal from FY 2020–2021 to FY 2022–2023 identified consistent high rates in Terai districts like Banke, Parsa, Bara, and Rautahat, where factors such as high population density, poverty and limited healthcare access likely contributed to the sustained spread of tuberculosis. In contrast, mountainous districts like Mustang, Kaski, and Solukhumbu consistently exhibited low TB prevalence, likely due to geographical isolation and lower population density. However, the increasing TB cases in previously lower-risk districts like Bhaktapur and Nuwakot in FY 2022–2023 emphasized the need for proactive healthcare interventions to prevent outbreaks. Many studies have employed Gi* statistics to detect spatial clusters of TB cases such as India [29], China [30, 31], Ethiopia [32] and Iran [33] which allowed for a more precise identification of hotspots. While our study identified high and low TB prevalence regions using spatial modeling techniques, incorporating Gi* statistics could further refine the detection of localized clusters. This approach would provide deeper insight into the spatial variations in TB spread and support a more targeted region-specific strategy for TB control in Nepal. The prevalence of TB in FY 2022–2023 decreased due to the COVID-19 pandemic with some cases going underreported. The finding is similar with this study reported that the pandemic disrupted TB prevention and control emphasizing the importance of maintaining essential health services and planning for recovery while considering the long-term effects on TB management [34].

Land surface temperature during daylight hours measures the heat radiated by the Earth's surface and is affected by factors such as solar radiation, surface composition, and land utilization. Elevated daytime temperatures might worsen respiratory problems, increasing persons' vulnerability to TB infection and potentially accelerating disease progression. The temperature could affect the human body’s physiological response to toxic agents and retard the clearance rate of *Mycobacterium tuberculosis* [35]. High temperatures can cause heat stress which compromises the immune system and facilitates the activation of latent TB infections in indirect ways [36, 37]. Within a specific range of air temperature and other environmental factors droplets containing *M. tuberculosis* are more prone to evaporating in the air and forming into specific diameters that can remain suspended for an extended period. Specifically, *M. tuberculosis* thrives and multiplies at temperatures between 35 and 37 °C and can survive for 4–5 years at temperatures ranging from −8 to −6 °C making these particles easily inhalable by susceptible individuals [38].

Our study found a significant association between LSTD, LSTN and the prevalence of TB aligning with previous research that links higher temperatures to increased TB transmission and prevalence. Nepal exhibits climatic diversity due to three distinct ecological belts, spanning from the mountainous region in the north to the flat terai region in the south. Therefore, we can witness several forms of climatic variation in Nepal. We hypothesized that various diseases, particularly infectious diseases such as TB are influenced by climatic conditions. Our recent study found that the land surface temperature throughout the day correlated with the prevalence of tuberculosis. Similarly, a previous study conducted in Nepal found a positive association between the incidence of TB and the land surface temperature at daylight and night [39].

A study from Ethiopia observed that high temperatures were associated with higher transmission rates of childhood TB in north-western Ethiopia suggesting that elevated temperatures can enhance the survival and transmission of *M. tuberculosis* in the environment [40]. Additionally, research by Brazil study indicated that TB was more prevalent at temperatures between 20 and 23 °C highlighting an optimal range for bacterial survival and transmission [41]. Studies have shown that higher temperatures are linked to increased TB notifications. Research in Japan also found that exposure to extreme heat significantly raised the risk of TB notifications while a separate study in Chinese mainland reported that even a one-degree rise in temperature increased the risk of TB notification [42, 43].

These findings suggested that higher temperatures can enhance *M. tuberculosis* survival, potentially increase indoor crowding to escape the heat and facilitate TB spread. Understanding this relationship is crucial for developing targeted public health interventions such as intensified TB screening and awareness campaigns during hotter periods, particularly in high-risk areas identified through spatial analysis. Furthermore, the interplay between environmental factors like temperature conditions must be considered.

The impact of precipitation on TB prevalence can be attributed to several underlying factors. A study from China highlighted that average annual rainfall directly indicates humidity levels had an inverse relationship with TB incidence rates [44]. A plausible explanation is that prolonged exposure to dry air can diminish the production of protective mucus on the respiratory tract's surface, thereby reducing resistance to *M. tuberculosis*. Laboratory studies have supported this, suggesting that dry conditions can impair the respiratory system's ability to prevent TB infection [45]. A study conducted on mice also discovered that the intake of dry air negatively impacted the ability of the airway cilia to sweep out foreign particles and impaired tissue repair activities. As a result, the body's ability to defend against viruses was diminished [46].

Our present study, combined with a review of previous research, emphasizes the complex relationship between precipitation and TB prevalence which varies across different geographic and climatic settings. A spatiotemporal analysis in Spain from 2012 to 2020 revealed a strong negative correlation between rainfall, sunshine, and TB incidence. This suggests that higher precipitation and sunlight may help reduce TB cases, potentially due to improved environmental conditions and the disinfecting effects of UV radiation [47]. Humidity and precipitation in the northwest areas of China with the arid conditions showed a negative association with tuberculosis cases. However, another area showed the opposite indicating that the regional condition affected the spread of TB [48]. The presence of precipitation was found to have a negative correlation with the prevalence of tuberculosis, potentially because rain tends to improve air quality and decrease the concentration of tuberculosis germs [49].

Urbanization is a key factor contributing to the high prevalence of TB due to several interrelated factors. The higher population density in urban areas facilitates TB transmission, as crowded living conditions make it easier for the bacteria to spread [50]. Additionally, urban environments often contain significant poverty and socioeconomic disparities limiting access to healthcare, nutritious food, and education about TB prevention [51]. Poor housing conditions, inadequate ventilation and poor sanitation heightened infection risks by compromising air quality especially for individuals from low-income families. Improving housing quality through rehousing or modifications enhanced respiratory health and reduced infection vulnerability [52]. The influx of migrants seeking better opportunities can introduce new TB cases while comorbidities like HIV/AIDS and diabetes in urban populations increase susceptibility [53]. Environmental factors, including pollution also negatively impact respiratory health [52]. Collectively, these elements underscored the need for targeted public health interventions in urban settings to combat TB effectively.

Cropland refers to areas used for agricultural production. Interestingly, despite the potential risks associated with farming, our study found a negative association between the extent of cropland and TB prevalence. The negative association between cropland areas and TB prevalence may relate to factors such as reduced population density and lower urbanization levels in agricultural regions rather than agricultural activity itself. Research indicated that urban areas, despite higher population density often have better healthcare access leading to earlier TB detection and reduced incidence whereas rural agricultural areas may have less access but lower transmission rates due to reduced density [54]. This finding suggested that regions with more agricultural land may experience lower TB rates, contrary to some prior studies. One possibility is that rural agricultural communities may have better overall air quality than urban areas as they are less exposed to industrial pollutants that can exacerbate respiratory conditions and potentially increase susceptibility to TB [55]. Additionally, the physical activity involved in farming might contribute to better overall health and stronger immune systems among farmers reducing their risk of TB.

The extent of agricultural land in a region can significantly influence the prevalence of TB due to various occupational and environmental factors. Farmers, predominantly located in areas with extensive cropland face a higher risk of TB infection due to several occupational hazards. Studies have shown a notable association between certain occupations, including farming, and increased TB rates [56]. Occupational exposure in farming can elevate TB risk due to prolonged exposure to dust, organic particles, and potentially lower access to healthcare facilities. A study from Ethiopia found that the prevalence of TB was higher among livestock farmers (59.7%), likely due to a lack of awareness about a type of tuberculosis infection, its transmission channels, and prevention methods [56]. In Nigeria, where 56.4% live below the poverty line and 90% reside in rural areas, the high number of farmers correlates with higher TB case rates, emphasizing the need for tailored interventions in farming communities [57].

Our study demonstrated a significant positive correlation between LSTN and urbanization with the prevalence of TB in Nepal. In FY 2020–2021, correlation coefficients of 1.53 for LSTN and 2.61 for urbanization indicated that higher levels of these factors were associated with increased TB prevalence. The Spatial Error Model (SEM) was the most suitable model for this period explaining 65.5% of the variance and showing positive autocorrelation between LSTN, urbanization, and TB prevalence, supported by AIC values of 695.49 for SEM and 697.326 for the Spatial Lag Model (SLM). In FY 2021–2022, the impact of LSTN and urbanization on TB prevalence remained significant with correlation coefficients of 1.21 for LSTN and 2.62 for urbanization. The SLM model was the most effective this year, accounting for 72.1% of the variance, as indicated by AIC values of 731.009 for SLM and 732.964 for SEM. By FY 2022–2023, LSTN and urbanization continued to significantly influence TB prevalence with correlation coefficients of 1.75 for LSTN and 2.64 for urbanization. The SLM model again provided the best fit explaining 69.6% of the variance. The AIC values showed that the SLM model outperformed the SEM with values of 724.45 for SLM and 726.924 for SEM.

These findings underscored the critical role of environmental factors, particularly LSTN and urbanization in influencing TB prevalence in Nepal across the studied fiscal years. The consistent performance of the SLM model indicates that spatial interactions between neighboring regions significantly impact TB transmission, highlighting the need for targeted public health interventions that consider these spatial dynamics. The increasing explained variance over the years suggests a growing complexity in the factors contributing to TB prevalence, likely influenced by changing environmental conditions and urbanization trends. Our findings are aligned with a study from Malaysia found that the Geographically Weighted Regression (GWR) model, compared to the OLS model, was the most effective in determining the spatial distribution of TB case with a high R^2^ value of 0.98. The GWR model’s local coefficient maps revealed that the influence of sociodemographic and environmental factors on TB incidence varied across different locations [58].

While this study employed advanced spatial analysis methodologies which provide a detailed understanding of the interactions between environmental variables and TB prevalence, certain limitations must be acknowledged. One significant drawback is the reliance on annual averages for temperature and precipitation which may obscure seasonal variations that could significantly influence TB transmission dynamics. Monthly or seasonal data would provide a more nuanced analysis, enabling better identification of patterns and correlations related to TB prevalence. Additionally, unmeasured confounding factors, such as housing conditions and socioeconomic disparities were not controlled for in this study. These factors can play a crucial role in shaping both environmental exposures and TB prevalence potentially confounding observed associations. Furthermore, the ecological fallacy must be considered as this study uses aggregate data that may not accurately reflect individual-level associations. The temporal scope of the analysis may also limit its ability to capture long-term trends or the effects of interventions, emphasizing the need for longitudinal studies to validate and expand upon these findings.

## Conclusions

This study highlighted the complex relationship between environmental factors, urbanization, and TB prevalence in Nepal. High TB prevalence in Terai districts such as Banke, Parsa, and Rautahat are linked to population density, poverty, and limited healthcare access. Therefore, proposal integrating climate data into public health monitoring systems is recommended. Significant correlations between LST and TB prevalence indicated that climate plays a crucial role in transmission, aligning with existing literature. Urbanization exacerbates TB risk due to socio-economic disparities and inadequate healthcare access. Overall, our findings underscored the need for targeted public health strategies considering environmental and socio-economic factors. Utilizing spatial modeling techniques can enhance the identification of high-risk areas, supporting tailored interventions to effectively combat TB in Nepal.

## Data Availability

The author has full access to the dataset. The electronic version of the data is stored at Khon Kaen University in Thailand. It will be made available upon request to anyone with an interest in the research topic. Readers who are interested are encouraged to contact the corresponding authors (alok.k@kkumail.com and suvash_ojha@swmu.edu.cn).

## Abbreviations

AIC: Akaike information criterion
AIDS: Acquired immunodeficiency syndrome
BIC: Bayesian information criterion
FY: Fiscal year
Gi statistics: *Getis-Ord statistic**
GWR: Geographically weighted regression
HIV: Human immunodeficiency virus
LISA: Local Indicators of Spatial Association
LSTD: Land surface temperature day
LSTN: Land surface temperature night
OLS: Ordinary least squares
QGIS: Quantum GIS
SEM: Spatial error model
SLM: Spatial lag model
TB: Tuberculosis
WGS: World geodetic system

## References

[1] Falzon D, Zignol M, Bastard M, Floyd K, Kasaeva T. The impact of the COVID-19 pandemic on the global tuberculosis epidemic. Front Immunol. 2023;14:1234785. 37795102 10.3389/fimmu.2023.1234785 PMC10546619

[2] WHO: Global Tuberculosis Report 2024. 2024.

[3] WHO: Global tuberculosis report 2023. 2023.

[4] Tuberculosis: Multidrug-resistant (MDR-TB) or rifampicin-resistant TB (RR-TB). https://www.who.int/news-room/questions-and-answers/item/tuberculosis-multidrug-resistant-tuberculosis-(mdr-tb). Accessed 20 May 2024.

[5] Bruchfeld J, Correia-Neves M, Källenius G. Tuberculosis and HIV coinfection. Cold Spring Harb Perspect Med. 2015;5(7):a017871. 25722472 10.1101/cshperspect.a017871 PMC4484961

[6] Bhatia V, Rijal S, Sharma M, Islam A, Vassall A, Bhargava A, et al. Ending TB in South-East Asia: flagship priority and response transformation. Lancet Reg Health Southeast Asia. 2023;18:100301. 38028166 10.1016/j.lansea.2023.100301 PMC10667305

[7] Organization WH: A situational analysis of programmatic management of TB preventive treatment in the WHO South-East Asia Region. 2020.

[8] Partnership S: Impact Assessment of Law, Human Rights, Gender, Key and Vulnerable Populations-related Barriers in Nepal's TB Response. 2022.

[9] Center NTC, Ministry of Health & Population N: National Tuberculosis Program Nepal (Annual Report). 2019.

[10] World Health Organization. WHO operational handbook on tuberculosis. Module 1: prevention-tuberculosis preventive treatment. Geneva: World Health Organization; 2020. https://www.who.int/publications/i/item/9789240002906.

[11] Putra IGNE, Rahmaniati M, Eryando T, Sipahutar T. Modeling the prevalence of tuberculosis in Java, Indonesia: an ecological study using geographically weighted regression. J Popul Soc Stud. 2022;30:741–63.

[12] Lin CH, Wen TH. How spatial epidemiology helps understand infectious human disease transmission. Trop Med Infect Dis. 2022;7(8):164. 36006256 10.3390/tropicalmed7080164 PMC9413673

[13] Fatima M, O’keefe KJ, Wei W, Arshad S, Gruebner O. Geospatial analysis of COVID-19: A scoping review. Int J Environ Res Public Health. 2021;18(5):2336. 33673545 10.3390/ijerph18052336 PMC7956835

[14] Sadovska D, Ozere I, Pole I, Ķimsis J, Vaivode A, Vīksna A, et al. Unraveling tuberculosis patient cluster transmission chains: integrating WGS-based network with clinical and epidemiological insights. Front Public Health. 2024;12:1378426. 38832230 10.3389/fpubh.2024.1378426 PMC11144917

[15] Shaweno D, Karmakar M, Alene KA, Ragonnet R, Clements ACA, Trauer JM, et al. Methods used in the spatial analysis of tuberculosis epidemiology: a systematic review. BMC Med. 2018;16:1–18. 10.1186/s12916-018-1178-4 PMC6193308 30333043

[16] Brown TS, Robinson DA, Buckee CO, Mathema B. Connecting the dots: understanding how human mobility shapes TB epidemics. Trends Microbiol. 2022;30(11):1036–44. 35597716 10.1016/j.tim.2022.04.005 PMC10068677

[17] Nepal Climate. https://weatherandclimate.com/nepal. Accessed 20 May 2024.

[18] Nepal. https://www.climatestotravel.com/temperatures/nepal. Accessed 20 May 2024.

[19] National Statistics Office (NSO) N: National Population and Housing Census 2021, 12th Population Census. 2021.

[20] Nepal Administrative Boundary (WGS 1984). https://download.hermes.com.np/nepal-administrative-boundary-wgs/. Accessed 25 May 2024.

[21] Anselin L, Syabri I, Kho Y. GeoDa: an introduction to spatial data analysis. In: Fischer MM, Getis A, editors. Handbook of applied spatial analysis. Berlin, Heidelberg: Springer; 2009. p. 73–89. 10.1007/978-3-642-03647-7_5.

[22] Getis A, Ord JK. The analysis of spatial association by use of distance statistics. Geogr Anal. 1992;24(3):189–206.

[23] Masrani AS, Nik Husain NR, Musa KI, Yasin AS. Trends and spatial pattern analysis of dengue cases in Northeast Malaysia. J Prev Med Public Health. 2022;55(1):80–7. 35135051 10.3961/jpmph.21.461 PMC8841195

[24] Anselin L. Local indicators of spatial association—LISA. Geogr Anal. 1995;27(2):93–115.

[25] Dismuke C, Lindrooth R. Ordinary least squares. Methods Des Outcomes Res. 2006;93(1):93–104.

[26] Rogerson P, Fotheringham AS, editors. The handbook of spatial analysis. Sage Publications; 2008. p. 528. https://www.torrossa.com/en/resources/an/4913744#page=270.

[27] Ward MD, Gleditsch KS. Spatial regression models, vol. 155. Sage Publications, Inc.; 2018. https://uk.sagepub.com/en-gb/eur/spatial-regression-models/book262155.

[28] Rajab NA, Hashim N, Rasam ARA. Spatial mapping and analysis of tuberculosis cases in Kuala lumpur, Malaysia. IEEE. 2020; 38–43.

[29] Rani R, Rameshwari T, Madaiah RK, Sunila, Kumar S. A Spatial analysis of Incidence of Tuberculosis infection using Interpolation Technique; 2018.

[30] Lin H, Zhang R, Wu Z, Li M, Wu J, Shen X, et al. Assessing the spatial heterogeneity of tuberculosis in a population with internal migration in China: a retrospective population-based study. Front Public Health. 2023;11:1155146. 37325311 10.3389/fpubh.2023.1155146 PMC10266412

[31] Alene KA, Xu Z, Bai L, Yi H, Tan Y, Gray D, et al. Spatial clustering of drug-resistant tuberculosis in Hunan province, China: an ecological study. BMJ Open. 2021;11(4):e043685. 33795303 10.1136/bmjopen-2020-043685 PMC8021748

[32] Alene KA, Viney K, Moore HC, Wagaw M, Clements ACA. Spatial patterns of tuberculosis and HIV co-infection in Ethiopia. PLoS ONE. 2019;14(12):e0226127. 31805149 10.1371/journal.pone.0226127 PMC6894814

[33] Kiani B, Raouf Rahmati A, Bergquist R, Hashtarkhani S, Firouraghi N, Bagheri N, et al. Spatio-temporal epidemiology of the tuberculosis incidence rate in Iran 2008 to 2018. BMC Public Health. 2021;21:1–20. 34098917 10.1186/s12889-021-11157-1 PMC8186231

[34] Alene KA, Wangdi K, Clements ACA. Impact of the COVID-19 pandemic on tuberculosis control: an overview. Trop Med Infect Dis. 2020;5(3):123. 32722014 10.3390/tropicalmed5030123 PMC7558533

[35] Niu Z, Qi Y, Zhao P, Li Y, Tao Y, Peng L, et al. Short-term effects of ambient air pollution and meteorological factors on tuberculosis in semi-arid area, northwest China: a case study in Lanzhou. Environ Sci Pollut Res Int. 2021;28(48):69190–9. 34291414 10.1007/s11356-021-15445-6

[36] Gao C, Wang Y, Hu Z, Jiao H, Wang L. Study on the associations between meteorological factors and the incidence of pulmonary tuberculosis in Xinjiang, China. Atmosphere. 2022;13(4):533.

[37] Li Z, Liu Q, Zhan M, Tao B, Wang J, Lu W. Meteorological factors contribute to the risk of pulmonary tuberculosis: a multicenter study in eastern China. Sci Total Environ. 2021;793:148621. 34328976 10.1016/j.scitotenv.2021.148621

[38] Rivas-Santiago CE, Sarkar S, Cantarella P IV, Osornio-Vargas Á, Quintana-Belmares R, Meng Q, et al. Air pollution particulate matter alters antimycobacterial respiratory epithelium innate immunity. Infect Immun. 2015;83(6):2507–17. 25847963 10.1128/IAI.03018-14 PMC4432745

[39] Sharma V, Laohasiriwong W, Mahato RK, Sornlorm K. Spatial association of socio-economic status and prevalence of Tuberculosis in Nepal, 2019. Int J Public Health Asia Pacific. 2022. 10.62992/ijphap.v1i1.15.

[40] Alene KA, Viney K, McBryde ES, Clements ACA. Spatiotemporal transmission and socio-climatic factors related to paediatric tuberculosis in north-western Ethiopia. Geospat Health. 2017;12(2):342–50. 10.4081/gh.2017.575 29239568

[41] Fernandes FMC, Martins ES, Pedrosa DMAS, Evangelista MSN. Relationship between climatic factors and air quality with tuberculosis in the Federal District, Brazil, 2003–2012. Brazil J Infect Dis. 2017;21:369–75. 10.1016/j.bjid.2017.03.017 PMC9428008 28545939

[42] Onozuka D, Hagihara A. The association of extreme temperatures and the incidence of tuberculosis in Japan. Int J Biometeorol. 2015;59:1107–14. 25351361 10.1007/s00484-014-0924-3

[43] Cao K, Yang K, Wang C, Guo J, Tao L, Liu Q, et al. Spatial-temporal epidemiology of tuberculosis in mainland China: an analysis based on Bayesian theory. Int J Environ Res Public Health. 2016;13(5):469. 27164117 10.3390/ijerph13050469 PMC4881094

[44] Xiao Y, He L, Chen Y, Wang Q, Meng Q, Chang W, et al. The influence of meteorological factors on tuberculosis incidence in Southwest China from 2006 to 2015. Sci Rep. 2018;8(1):10053. 29968800 10.1038/s41598-018-28426-6 PMC6030127

[45] Fahy JV, Dickey BF. Airway mucus function and dysfunction. N Engl J Med. 2010;363(23):2233–47. 21121836 10.1056/NEJMra0910061 PMC4048736

[46] Kudo E, Song E, Yockey LJ, Rakib T, Wong PW, Homer RJ, et al. Low ambient humidity impairs barrier function and innate resistance against influenza infection. Proc Natl Acad Sci U S A. 2019;116(22):10905–10. 31085641 10.1073/pnas.1902840116 PMC6561219

[47] Galán MMD, Redondo-Bravo L, Gómez-Barroso D, Herrera L, Amillategui R, Gómez-Castellá J, et al. The impact of meteorological factors on tuberculosis incidence in Spain: a spatiotemporal analysis. Epidemiol Infect. 2024;152:e58. 38505884 10.1017/S0950268824000499 PMC11022253

[48] Li H, Ge M, Zhang MX. Spatio-temporal distribution of tuberculosis and the effects of environmental factors in China. BMC Infect Dis. 2022;22(1):565. 35733132 10.1186/s12879-022-07539-4 PMC9215012

[49] Wang X, Yin S, Li Y, Wang W, Du M, Guo W, et al. Spatiotemporal epidemiology of, and factors associated with, the tuberculosis prevalence in northern China, 2010–2014. BMC Infect Dis. 2019;19:365. 10.1186/s12879-019-3910-x. 10.1186/s12879-019-3910-x PMC6492399 31039734

[50] Harling G, Castro MC. A spatial analysis of social and economic determinants of tuberculosis in Brazil. Health Place. 2014;25:56–67. 24269879 10.1016/j.healthplace.2013.10.008

[51] Vilar-Compte M, Burrola-Méndez S, Lozano-Marrufo A, Ferré-Eguiluz I, Flores D, Gaitán-Rossi P, et al. Urban poverty and nutrition challenges associated with accessibility to a healthy diet: a global systematic literature review. Int J Equity Health. 2021;20(1):40. 33472636 10.1186/s12939-020-01330-0 PMC7816472

[52] Holden KA, Lee AR, Hawcutt DB, Sinha IP. The impact of poor housing and indoor air quality on respiratory health in children. Breathe (Sheff). 2023;19(2):230058. 37645022 10.1183/20734735.0058-2023 PMC10461733

[53] Hayward S, Harding RM, McShane H, Tanner R. Factors influencing the higher incidence of tuberculosis among migrants and ethnic minorities in the UK. F1000Res. 2018;7:461. 30210785 10.12688/f1000research.14476.1 PMC6107974

[54] Liyew AM, Clements ACA, Akalu TY, Gilmour B, Alene KA. Ecological-level factors associated with tuberculosis incidence and mortality: a systematic review and meta-analysis. PLOS Global Public Health. 2024;4(10):e0003425. 39405319 10.1371/journal.pgph.0003425 PMC11478872

[55] Xiang K, Xu Z, Hu YQ, He YS, Dan YL, Wu Q, et al. Association between ambient air pollution and tuberculosis risk: a systematic review and meta-analysis. Chemosphere. 2021;277:130342. 33794431 10.1016/j.chemosphere.2021.130342

[56] Adane A, Damena M, Weldegebreal F, Mohammed H. Prevalence and associated factors of tuberculosis among adult household contacts of smear positive pulmonary tuberculosis patients treated in public health facilities of Haramaya District, Oromia Region, Eastern Ethiopia. Tuberc Res Treat. 2020;2020:6738532. 32047665 10.1155/2020/6738532 PMC7007743

[57] Milaham M, Van Gurp M, Adewusi OJ, Okonuga OC, Ormel H, Tristan B, et al. Assessment of tuberculosis case notification rate: spatial mapping of hotspot, coverage and diagnostics in Katsina State, north-western Nigeria. J Public Health Afr. 2022;13(3):2040. 36337675 10.4081/jphia.2022.2040 PMC9627762

[58] Mohidem NA, Osman M, Hashim Z, Muharam FM, Mohd Elias S, Shaharudin R. Association of sociodemographic and environmental factors with spatial distribution of tuberculosis cases in Gombak, Selangor, Malaysia. PLoS ONE. 2021;16(6):e0252146. 34138899 10.1371/journal.pone.0252146 PMC8211220

